# Camizestrant in Combination with Three Globally Approved CDK4/6 Inhibitors in Women with ER+, HER2− Advanced Breast Cancer: Results from SERENA-1

**DOI:** 10.1158/1078-0432.CCR-25-1198

**Published:** 2025-08-11

**Authors:** Richard D. Baird, Begoña Bermejo de las Heras, Manuel Ruiz-Borrego, Christos Vaklavas, Irene Moreno, Mafalda Oliveira, Anne Armstrong, Nicholas Turner, Jason Incorvati, Chris Twelves, Eva Ciruelos, Erika Hamilton, Manish R. Patel, Peter Kabos, Carmela Ciardullo, Teresa Klinowska, Justin P.O. Lindemann, Alastair M. Mathewson, Christopher J. Morrow, Andy Sykes, Jincheng Yang, Bairu Zhang, Ivan Victoria

**Affiliations:** 1 Cancer Research UK, Cambridge Centre, Cambridge, United Kingdom.; 2 Department of Medical Oncology, Hospital Clinico Universitario de Valencia, Valencia, Spain.; 3 Department of Medical Oncology, H U Virgen del Rocio, Seville, Spain.; 4 Huntsman Cancer Institute, University of Utah, Salt Lake City, Utah.; 5 START Madrid-CIOCC, Centro Integral Oncológico Clara Campal, Madrid, Spain.; 6 Medical Oncology Department, Vall d’Hebron University Hospital and Breast Cancer Group, Vall d’Hebron Institute of Oncology, Barcelona, Spain.; 7 The Christie NHS Foundation Trust and the University of Manchester, Manchester, United Kingdom.; 8 Cancer Now, Toby Robins Research Centre, Institute of Cancer Research, London, United Kingdom.; 9 Fox Chase Cancer Center, Philadelphia, Pennsylvania.; 10 University of Leeds Teaching Hospitals NHS Trust, Leeds, United Kingdom.; 11 Medical Oncology Department, University Hospital 12 de Octubre, Madrid, Spain.; 12 Sarah Cannon Research Institute, Nashville, Tennessee.; 13 Florida Cancer Specialists/Sarasota Memorial Hospital, Sarasota, Florida.; 14 Division of Medical Oncology, University of Colorado, Boulder, Colorado.; 15 Research and Early Development, Oncology R&D, AstraZeneca, Cambridge, United Kingdom.; 16 Late Development, Oncology R&D, AstraZeneca, Cambridge, United Kingdom.; 17 Clinical Pharmacology and Safety Sciences, Biopharmaceutical Sciences, AstraZeneca, Cambridge, United Kingdom.; 18 Research and Early Development, Oncology R&D, AstraZeneca, Waltham, Massachusetts.; 19 Department of Medical Oncology, IDIBAPS, Hospital Clínic, Barcelona, Spain.

## Abstract

**Purpose:**

This trial investigated the safety and tolerability of camizestrant with cyclin-dependent kinase 4/6 inhibitors (CDK4/6i) in women with estrogen receptor–positive, HER2− advanced breast cancer.

**Patients and Methods:**

SERENA-1 (NCT03616587) is a phase I, multipart, open-label study in women with refractory estrogen receptor–positive, HER2− advanced breast cancer. Patients received oral once-daily camizestrant 75 or 150 mg plus abemaciclib; camizestrant 75, 150, or 300 mg plus palbociclib; or camizestrant 75 mg plus ribociclib 400 or 600 mg. Safety/tolerability, pharmacokinetics, efficacy, and impact on estrogen receptor 1 mutation ctDNA were assessed.

**Results:**

By September 16, 2024 (data cutoff), 53 patients had received camizestrant plus abemaciclib, 78 camizestrant plus palbociclib, and 60 camizestrant plus ribociclib. Patients had a median of 2 (range, 0–7) prior regimens for advanced disease; 83% had received a prior CDK4/6i and 59% prior fulvestrant. The most common treatment-emergent adverse events for camizestrant 75 mg (phase III dose) plus each CDK4/6i were diarrhea [with abemaciclib (87.5%)] and neutropenia [with palbociclib (80%) and ribociclib (32.1% for 400 mg and 53.1% for 600 mg)]. The median camizestrant t_max_ was ∼4 hours postdose across combinations, with an estimated half-life of 9.5 to 17 hours. No clinically meaningful drug–drug interactions were evident. In this heavily pretreated population, CBR_24_ was 49.5% and the median progression-free survival was 7.4 months (95% confidence interval, 5.3–9.3), with antitumor activity across all combinations, including patients previously treated with CDK4/6i and/or fulvestrant, with or without estrogen receptor 1 mutation.

**Conclusions:**

Camizestrant is well tolerated, with antitumor activity in combination with CDK4/6i. These results support the evaluation of camizestrant 75 mg plus standard CDK4/6i doses in phase III trials.

**Translational Relevance**

Estrogen receptor (ER) signaling is a key therapeutic target in ER+, HER2− breast cancer. Current endocrine therapies in combination with CDK4/6 inhibitors (CDK4/6i) have proved efficacy in both early and advanced disease, although resistance to these treatments remains a significant clinical challenge. Camizestrant, the next-generation oral selective estrogen receptor degrader and complete ER antagonist, is in phase III development for the treatment of ER+/HER2− breast cancer, as monotherapy and in combination with CDK4/6i. In this phase I open-label, multipart SERENA-1 trial, camizestrant 75 mg, received once daily (the phase III dose) in combination with abemaciclib, palbociclib, or ribociclib, demonstrated a well-tolerated safety profile, no clinically meaningful drug–drug interactions, and encouraging clinical activity in this heavily pretreated population including prior CDK4/6i, fulvestrant, and chemotherapy.

## Introduction

Breast cancer is the second most common cancer globally, with more than 2.3 million new cases reported in 2022 alone (1). Hormone receptor (HR)–positive breast cancer, including estrogen receptor (ER)–positive disease, is the most common type, accounting for approximately 68% to 75% of cases (2–4).

Endocrine therapy (ET), which interferes with ER signaling, is the backbone of treatment for HR+ breast cancer (5). However, despite the standard therapy, relapse occurs in many patients (6), highlighting the need for more effective targeted therapies.

Selective ER degraders (SERD) are a type of ET that targets the ER directly, resulting in its antagonism and degradation (7). Fulvestrant was the first SERD approved as a monotherapy for HR+ breast cancer (8) and is also indicated for the treatment of HR+, HER2− breast cancer in combination with the approved cyclin-dependent kinase 4/6 inhibitors (CDK4/6i) abemaciclib (6), palbociclib (7), and ribociclib (9). All three of these CDK4/6i have been shown to be highly effective in combination with ET in early (9, 10) and/or advanced (11–24) disease. However, differences in the pharmacology and pharmacokinetic (PK) profiles of these agents contribute to important variations in their dosing schedules, potential for drug–drug interactions, and safety profiles (25, 26).

Camizestrant, the next-generation oral SERD and complete ER antagonist, is currently in phase III development for HR+, HER2− breast cancer (27–30). In preclinical studies, camizestrant has demonstrated potent ER degradation, with no evidence of agonism, and robust antitumor effects in both estrogen receptor 1 (*ESR1*) wild-type and mutant models (31). SERENA-1 (NCT03616587) is a multipart, phase I, first-in-human, dose escalation, and expansion study designed to evaluate the safety and tolerability of camizestrant as a monotherapy and in combination with other targeted anticancer agents in women with ER+, HER2− advanced breast cancer. Camizestrant monotherapy results (parts A and B) from SERENA-1 have been reported previously and demonstrated the preliminary safety, efficacy, and PK profile of camizestrant, with dose-dependent exposures suggesting a half-life of 20 to 23 hours (32). In this study, 75, 150, and 300 mg once-daily doses were selected for phase II testing. The safety and efficacy of camizestrant monotherapy have also been evaluated in the phase II SERENA-2 study, in which camizestrant demonstrated progression-free survival (PFS) superiority over fulvestrant in patients with pretreated ER+, HER2− advanced breast cancer (33). Furthermore, the phase III SERENA-6 trial (NCT04964934) met its primary endpoint, in which switching to camizestrant with continuation of CDK4/6i guided by the emergence of *ESR1* mutation (*ESR1*m) during first-line therapy, ahead of disease progression, demonstrated statistically significant and clinically meaningful improvement in PFS versus continuing aromatase inhibitor + CDK4/6i in patients with HR+, HER2− advanced breast cancer (34).

Here, we describe the results from SERENA-1 cohorts, which investigated safety and tolerability, PK, pharmacodynamics, and efficacy of camizestrant in combination with abemaciclib (parts G/H), palbociclib (parts C/D), and ribociclib (parts K/L).

## Patients and Methods

### Study overview

Details of the study’s key design elements, eligibility criteria, endpoints, assessments, compliance, and oversight have previously been published (35).

All participants consented to participate in this study and gave their written informed consent prior to enrollment. The study was performed in line with the principles of the Declaration of Helsinki, Council for International Conference on Harmonization Guidelines for Good Clinical Practice, and all applicable national and local laws. The protocol was approved by the respective regulatory authorities and the research ethics committee of each participating site and was subject to ethics committee and institutional review board approvals.

Briefly, participants were recruited from 16 sites in the United Kingdom, Spain, and the United States. Pre- or postmenopausal women with metastatic or recurrent ER+, HER2− adenocarcinoma of the breast were eligible. Patients must have received prior treatment with ≥1 ET, but ≤2 lines of chemotherapy, in the advanced/metastatic setting. Prior treatment with CDK4/6i was permitted.

### Objectives

The primary objective of SERENA-1 was to investigate the safety and tolerability of camizestrant alone and in combination with other ETs in women with ER+, HER2− advanced breast cancer and to define dosing regimens for further clinical evaluation.

Secondary objectives included the assessment of efficacy, antitumor activity, and PK of camizestrant alone and in combination with other ETs.

### Study design

All CDK4/6i were administered as per their relevant regional labels for advanced disease, except for ribociclib, where doses recommended for both early (400 mg) and advanced (600 mg) disease were evaluated. Using a dose-escalation/expansion format [details published previously (36)], patients were enrolled onto the study to receive oral once-daily camizestrant 75 or 150 mg plus abemaciclib; camizestrant 75, 150, or 300 mg plus palbociclib; or camizestrant 75 mg plus ribociclib 400 or 600 mg. Dosing schedules, safety assessments, and dose interruptions and reductions for each CDK4/6i were in accordance with their labels. Adjustment of the initial CDK4/6i dose was permitted based on whether the patient was previously treated with the allocated CDK4/6i.

Details of safety and efficacy assessments and of ctDNA sampling and assessments have been described previously (37). For PK analyses, plasma concentrations of camizestrant, abemaciclib, palbociclib, and ribociclib were determined using validated LC/MS-MS.

### Statistical analysis

The study schema (and patient disposition) of the reported cohorts is provided in Supplementary Fig. S1. For each study part, target enrollment was up to 12 patients per arm for the dose-escalation phase, followed by up to 12 additional patients per arm in the dose-expansion phase.

Details of assessments and analysis sets have been described previously (38). Briefly, safety was assessed in the safety analysis set, and efficacy and antitumor activity were assessed in the evaluable-for-response set. PK was assessed in the PK analysis set, which included all patients who provided at least one quantifiable postdose PK concentration for either camizestrant or any of the other agents evaluated.

Descriptive statistics were used for all variables. PFS was assessed using the Kaplan–Meier method. Unless otherwise stated, percentages were calculated from the analysis set in total and for each cohort, or combined cohorts where appropriate.

## Results

Enrollment occurred sequentially for all parts between March 10, 2021, and July 17, 2023, for the camizestrant plus abemaciclib group; September 19, 2019, and November 23, 2020, for camizestrant plus palbociclib; and February 28, 2023, and January 11, 2024, for camizestrant plus ribociclib. The data cutoff was September 16, 2024, for camizestrant plus abemaciclib or ribociclib and September 9, 2021, for camizestrant plus palbociclib. Overall, 191 patients were enrolled and treated: 53 with camizestrant plus abemaciclib, 78 with camizestrant plus palbociclib and 60 with camizestrant plus ribociclib.

### Baseline characteristics

Baseline characteristics were broadly consistent across all parts (Table 1). Overall, patients were heavily pretreated, with a median of 2 (0–7) prior lines of therapy in the advanced setting. Similar numbers of patients in each part received prior chemotherapy or ET in the advanced disease setting. Around half (59%) of participants had received prior fulvestrant, and 83% had received prior CDK4/6i in the advanced disease. Patient disposition is described in Supplementary Fig. S1. Overall representativeness of the trial is reported in Supplementary Table S1 (1–4, 39–42).

**Table 1.** Baseline characteristics and patient demographics for camizestrant in combination with abemaciclib, palbociclib, or ribociclib.

Characteristic	C 75 mg + A (*n* = 24)	C 150 mg + A *(n* = 29)	C 75 mg + P (*n* = 25)	C 150 mg + P (*n* = 24)	C 300 mg + P (*n* = 29)	C 75 mg + R 400 mg (*n* = 28)	C 75 mg + R 600 mg (*n* = 32)
Median age, years (range)	61 (39–89)	60 (31–83)	58 (47–77)	59 (39–76)	58 (34–76)	58 (31–81)	55 (37–80)
Postmenopausal, *n* (%)	22 (92)	25 (86)	25 (100)	24 (100)	25 (86)	24 (86)	27 (84)
ECOG category 0, *n* (%)	11 (46)	15 (52)	10 (40.0)	11 (46)	16 (55)	14 (50)	17 (53)
Measurable disease, *n* (%)	19 (79)	22 (76)	17 (68.0)	19 (79)	26 (90)	18 (64)	24 (75)
Visceral disease, *n* (%)	16 (67)	21 (72)	16 (64.0)	18 (75)	23 (79)	20 (71)	22 (69)
Liver visceral disease, *n* (%)	13 (54)	15 (52)	13 (52)	12 (50)	16 (55)	18 (64)	18 (56)
Lung visceral disease, *n* (%)	6 (25)	9 (31)	9 (36)	11 (46)	14 (48)	8 (29)	8 (25)
Liver and lung visceral disease, *n* (%)	3 (13)	5 (17)	7 (28)	5 (21)	9 (31)	6 (21)	4 (13)
Number of prior regimens in the advanced setting, median (range)	3 (1–7)	2 (1–4)	2 (1–5)	3 (1–7)	2 (1–6)	2 (0–5)	2 (1–6)
Number of prior endocrine regimens in the advanced setting, median (range)^a^	2 (1–5)	1 (0–4)	2 (0–4)	2 (0–5)	2 (1–4)	2 (0–4)	2 (0–5)
Number of prior chemotherapy regimens in the advanced setting, median (range)	0 (0–2)	0 (0–2)	0 (0–2)	1 (0–2)	1 (0–2)	0 (0–1)	1 (0–2)
Prior chemotherapy in the advanced setting, *n* (%)	11 (46)	8 (28)	12 (48)	14 (58)	15 (52)	7 (25)	17 (53)
Prior treatment with fulvestrant in the advanced setting, *n* (%)	14 (58)	12 (41)	17 (68)	15 (63)	16 (55)	17 (61)	21 (65.6)
Prior treatment with CDK4/6i in the advanced setting, *n* (%)^b^	19 (79)	25 (86)	20 (80)	16 (67)	21 (72)	25 (89)	32 (100)
Palbociclib	16 (67)	17 (59)	16 (64)	13 (54)	13 (45)	13 (46)	17 (53)
Abemaciclib	0	2 (7)	3 (12)	1 (4)	5 (17)	5 (18)	8 (25)
Ribociclib	3 (13)	6 (21)	3 (12)	2 (8)	6 (21)	9 (32)	9 (28)
*ESR1*m detected at baseline, *n* (%)	​	​	​	​	​	​	​
Detected	11 (46)	16 (55)	11 (44)	16 (67)	12 (41)	16 (57)	12 (38)
Not detected	12 (50)	13 (45)	14 (56)	8 (33)	13 (45)	12 (43)	20 (63)
Unknown	1 (4)	0	0	0	4 (14)	0	0

Abbreviations: A, abemaciclib; C, camizestrant; ECOG, Eastern Cooperative Oncology Group; P, palbociclib; R, ribociclib.

a Includes eight patients who either did not meet the inclusion criterion for prior ET in the advanced setting or were incorrectly recorded in the case report form as has having received ET in the early setting when they had received it in the advanced setting.

b Patients may have received more than one prior CDK4/6i.

### Safety

The mean treatment duration in months (± SD) and safety summary for all combinations are shown in Supplementary Table S2. The most common treatment-emergent adverse events (TEAE) of any grade for camizestrant (75 and 150 mg) plus abemaciclib were diarrhea (87.5% and 82.8%) and nausea (45.8% and 44.8%), and the most common grade ≥3 TEAE was neutrophil count decreased (16.7% and 13.8%). For camizestrant (75, 150, and 300 mg) plus palbociclib, neutropenia was both the most common TEAE of any grade (80.0%, 83.3%, and 75.9%) and the most common grade ≥3 TEAE (56.0%, 66.7%, and 62.1%). For camizestrant (75 mg) plus ribociclib (400 and 600 mg), the most common TEAEs of any grade were neutropenia (32.1% and 53.1%) and nausea (35.7% and 43.8%), and the most common grade ≥3 TEAE was neutropenia (10.7% and 43.8%; Table 2; Supplementary Table S3). No instances of febrile neutropenia were reported in any of the combinations.

**Table 2.** AE profile for camizestrant in combination with abemaciclib, palbociclib, or ribociclib (irrespective of causality); AEs reported in ≥20% of patients.^a^

*N* (%)	C 75 mg + A (*n* = 24)		C 150 mg + A (*n* = 29)		C 75 mg + P (*n* = 25)		C 150 mg + P (*n* = 24)		C 300 mg + P (*n* = 29)		C 75 mg + R 400 mg (*n* = 28)		C 75 mg + R 600 mg (*n* = 32)	
	Any grade	Grade ≥3	Any grade	Grade ≥3	Any grade	Grade ≥3	Any grade	Grade ≥3	Any grade	Grade ≥3	Any grade	Grade ≥3	Any grade	Grade ≥3
Abdominal pain	5 (20.8)	1 (4.2)	5 (17.2)	0	2 (8.0)	1 (4.0)	5 (20.8)	0	2 (6.9)	0	5 (17.9)	1 (3.6)	1 (3.1)	0
Alanine aminotransferase increased	5 (20.8)	3 (12.5)	4 (13.8)	3 (10.3)	0	0	1 (4.2)	0	2 (6.9)	0	2 (7.1)	0	5 (15.6)	1 (3.1)
Anemia	4 (16.7)	0	8 (27.6)	2 (6.9)	5 (20.0)	1 (4.0)	8 (33.3)	1 (4.2)	9 (31.0)	1 (3.4)	5 (17.9)	0	4 (12.5)	0
Arthralgia	6 (25.0)	0	1 (3.4)	0	3 (12.0)	0	2 (8.3)	0	6 (20.7)	0	7 (25.0)	0	4 (12.5)	1 (3.1)
Aspartate aminotransferase increased	6 (25.0)	1 (4.2)	6 (20.7)	3 (10.3)	0	0	4 (16.7)	0	1 (3.4)	0	3 (10.7)	0	6 (18.8)	1 (3.1)
Asthenia	6 (25.0)	1 (4.2)	9 (31.0)	0	3 (12.0)	0	8 (33.3)	0	10 (34.5)	0	8 (28.6)	0	6 (18.8)	0
Back pain	5 (20.8)	0	6 (20.7)	1 (3.4)	1 (4.0)	1 (4.0)	2 (8.3)	0	4 (13.8)	0	2 (7.1)	0	5 (15.6)	1 (3.1)
Blood creatinine increased	6 (25.0)	0	9 (31.0)	1 (3.4)	2 (8.0)	0	1 (4.2)	0	2 (6.9)	0	4 (14.3)	0	4 (12.5)	0
Bradycardia	2 (8.3)	0	5 (17.2)	0	0	0	4 (16.7)	0	7 (24.1)	0	1 (3.6)	0	2 (6.3)	0
Constipation	2 (8.3)	0	7 (24.1)	0	3 (12.0)	0	5 (20.8)	0	4 (13.8)	0	1 (3.6)	0	2 (6.3)	0
Cough	9 (37.5)	0	4 (13.8)	0	0	0	4 (16.7)	0	2 (6.9)	0	7 (25.0)	0	6 (18.8)	0
COVID-19	6 (25.0)	0	2 (6.9)	0	0	0	1 (4.2)	1 (4.2)	2 (6.9)	0	2 (7.1)	0	1 (3.1)	0
Decreased appetite	9 (37.5)	0	7 (24.1)	1 (3.4)	3 (12.0)	0	4 (16.7)	1 (4.2)	1 (3.4)	0	2 (7.1)	0	1 (3.1)	0
Diarrhea	21 (87.5)	2 (8.3)	24 (82.8)	2 (6.9)	3 (12.0)	0	1 (4.2)	0	1 (3.4)	0	8 (28.6)	2 (7.1)	8 (25.0)	0
Dizziness	6 (25.0)	0	3 (10.3)	2 (6.9)	2 (8.0)	0	4 (16.7)	0	2 (6.9)	0	2 (7.1)	0	5 (15.6)	0
Dry eye	5 (20.8)	0	3 (10.3)	0	1 (4.0)	0	3 (12.5)	0	6 (20.7)	0	1 (3.6)	0	2 (6.3)	0
Dysgeusia	5 (20.8)	1 (4.2)	5 (17.2)	0	2 (8.0)	0	1 (4.2)	0	1 (3.4)	0	0	0	0	0
Dyspnea	2 (8.3)	0	5(17.2)	2 (6.9)	2 (8.0)	0	3 (12.5)	1 (4.2)	1 (3.4)	0	7 (25.0)	1 (3.6)	5 (15.6)	1 (3.1)
QT prolonged	0	0	3 (10.3)	2 (6.9)	1 (4.0)	0	1 (4.2)	0	2 (6.9)	0	6 (21.4)	1 (3.6)	5 (15.6)	2 (6.3)
Fatigue	11 (45.8)	2 (8.3)	8 (27.6)	0	5 (20.0)	0	6 (25.0)	1 (4.2)	5 (17.2)	0	5 (17.9)	0	7 (21.9)	0
Hemoglobin decreased	2 (8.3)	0	6 (20.7)	0	0	0	0	0	0	0	3 (10.7)	0	5 (15.6)	0
Headache	7 (29.2)	0	6 (20.7)	0	2 (8.0)	0	1 (4.2)	0	2 (6.9)	0	3 (10.7)	0	0	0
Hypertension	5 (20.8)	3 (12.5)	3 (10.3)	0	2 (8.0)	2 (8.0)	0	0	1 (3.4)	0	5 (17.9)	1 (3.6)	5 (15.6)	0
Lacrimation increased	5 (20.8)	0	0	0	0	0	1 (4.2)	0	1 (3.4)	0	0	0	0	0
NT-proBNP increased	2 (8.3)	0	5 (17.2)	0	0	0	0	0	1 (3.4)	0	2 (7.1)	0	7 (21.9)	0
Nausea	11 (45.8)	0	13 (44.8)	1 (3.4)	4 (16.0)	0	7 (29.2)	1 (4.2)	7 (24.1)	0	10 (35.7)	1 (3.6)	14 (43.8)	0
Neutropenia	7 (29.2)	5 (20.8)	6 (20.7)	1 (3.4)	20 (80.0)	14 (56.0)	20 (83.3)	16 (66.7)	22 (75.9)	18 (62.1)	9 (32.1)	3 (10.7)	17 (53.1)	14 (43.8)
Neutrophil count decreased	6 (25.0)	4 (16.7)	5 (17.2)	4 (13.8)	0	0	0	0	0	0	4 (14.3)	2 (7.1)	5 (15.6)	5 (15.6)
Photopsia	4 (16.7)	0	6 (20.7)	0	4 (16.0)	0	6 (25.0)	0	6 (20.7)	0	11 (39.3)	0	11 (34.4)	0
Platelet count decreased	6 (25.0)	0	2 (6.9)	1 (3.4)	4 (16.0)	0	5 (20.8)	2 (8.3)	8 (27.6)	4 (13.8)	0	0	0	0
Pruritus	5 (20.8)	0	0	0	1 (4.0)	0	1 (4.2)	0	1 (3.4)	0	4 (14.3)	0	2 (6.3)	0
Sinus bradycardia	5 (20.8)	0	12 (41.4)	0	4 (16.0)	0	5 (20.8)	0	12 (41.4)	0	11 (39.3)	0	10 (31.3)	0
Urinary tract infection	8 (33.3)	0	6 (20.7)	0	2 (8.0)	0	3 (12.5)	0	2 (6.9)	0	3 (10.7)	0	2 (6.3)	0
Visual impairment	7 (29.2)	0	9 (31.0)	0	5 (20.0)	0	8 (33.3)	0	5 (17.2)	0	5 (17.9)	0	4 (12.5)	0
Vitreous floaters	1 (4.2)	0	3 (10.3)	0	0	0	2 (8.3)	0	0	0	2 (7.1)	0	7 (21.9)	0
Vomiting	8 (33.3)	0	11 (37.9)	1 (3.4)	3 (12.0)	0	6 (25.0)	0	3 (10.3)	0	6 (21.4)	1 (3.6)	7 (21.9)	0
Weight decreased	5 (20.8)	0	3 (10.3)	1 (3.4)	0	0	1 (4.2)	0	0	0	0	0	0	0

Common Terminology Criteria for Adverse Events grades are reported as the number of patients with grade 1, grade 2, grade 3, grade 4, and grade 5. All data are presented as *N* (%).

Abbreviations: A, abemaciclib; C, camizestrant; NT-proBNP, N-terminal prohormone brain natriuretic peptide; P, palbociclib; R, ribociclib.

a Reported in ≥20% of patients in any cohort at any grade.

**Figure 1.**
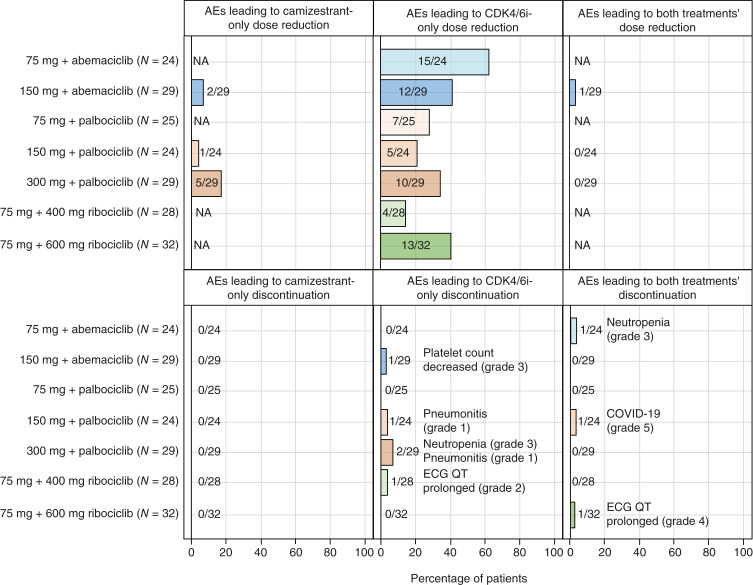
AEs leading to dose reduction or discontinuation in patients treated with camizestrant 75, 150, or 300 mg combined with either abemaciclib, palbociclib, or ribociclib. Description data are presented as number and % of patients. N, number; NA, not applicable.

There were no reports of adverse events (AE) leading to discontinuation of camizestrant only (Fig. 1). In total, three patients discontinued both camizestrant and CDK4/6i because of AEs; one patient discontinued both camizestrant 150 mg and palbociclib following COVID-19 (grade 5); one discontinued both camizestrant 75 mg and abemaciclib because of neutropenia (grade 3), and one patient discontinued both camizestrant 75 mg and ribociclib 600 mg because of QT prolongation (grade 4).

In line with previous studies for the phase III dose of camizestrant monotherapy at 75 mg, AEs of photopsia and sinus bradycardia, predominantly grade 1, were observed across all arms (Table 2). For camizestrant 75 mg combinations with CDK4/6i, photopsia was reported in 27.5% (30/109) of patients; all were grade 1 with no impact on activities of daily living for any patient. A total of 51.4% (56/109) of patients reported a vision-related AE, with a median onset of 7 days. If experienced, these AEs were described as intermittent, short-lived, and not requiring intervention. Of those reporting these AEs, 64.3% (36/56) of patients reported resolution either during [33.9% (19/56)] or shortly following cessation [30.4% (17/56)] of camizestrant; 19.6% (11/56) of patients were still receiving camizestrant treatment at data cutoff with ongoing visual effects. Ophthalmologic review of all patients showed no evidence of structural or retinal changes to the eye.

AEs of sinus bradycardia were all grade 1 (asymptomatic) for the camizestrant 75 mg combinations, except for one grade 2 in camizestrant plus ribociclib 400 mg.

Digital, centrally read, triplicate ECGs were obtained throughout the study. Camizestrant treatment was associated with a dose- and time-dependent reversible reduction in the resting heart rate (HR), with a gradual decrease to a stable nadir over approximately 14 days, while maintaining sinus rhythm. Reversion to baseline resting HR following cessation of dosing had a profile that was broadly symmetrical to that at onset. Treatment with camizestrant 75 mg was associated with a median change in HR nadir on cycle 1, day 15 predose versus baseline of −12.3 bpm (IQR: −21.7, −8.0) when combined with abemaciclib; −19.7 bpm (IQR: −24.3, −8.83) when combined with palbociclib; and −19.2 bpm (IQR: −24.7, −13.3) when combined with ribociclib {Supplementary Fig. S2A [mean (±SD) HR also reported]}.

Adverse events of QTcF prolongation in the 75 mg combination arms were reported in 11.0% (12/109) of participants overall: 0% (0/24), 4% (1/25), and 18.3% (11/60) for camizestrant plus abemaciclib, palbociclib, and ribociclib, respectively. There were three instances of grade ≥3 QTcF prolongation across all combinations. The median change in QTcF from baseline to cycle 1, day 15 dose (derived from centrally read ECGs) was 7.7 ms (IQR: −3.0, 17.0) for camizestrant plus abemaciclib, 8.7 ms (IQR: 3.7, 18.3) for camizestrant plus palbociclib, and 28.0 ms (IQR: 13.3, 33.0) for camizestrant plus ribociclib (Supplementary Fig. S2B).

### PK

After multiple dosing (cycle 1, day 15) of camizestrant in combination with each CDK4/6i, the median t_max_ for camizestrant was approximately 4 hours after dose across the dose range (Supplementary Fig. S3; Supplementary Table S4).

Camizestrant plasma PK on cycle 1, day 15 in combination with either palbociclib or abemaciclib were comparable with camizestrant monotherapy PK. When camizestrant 75 mg was dosed with ribociclib 400 or 600 mg, the exposure of camizestrant was increased and more in line with the exposure of camizestrant 150 mg as monotherapy.

PK profiles for palbociclib, abemaciclib, and ribociclib were as expected based on population PK simulations for each drug individually (Supplementary Fig. S3; refs. 35, 38).

### Antitumor activity

**Figure 2.**
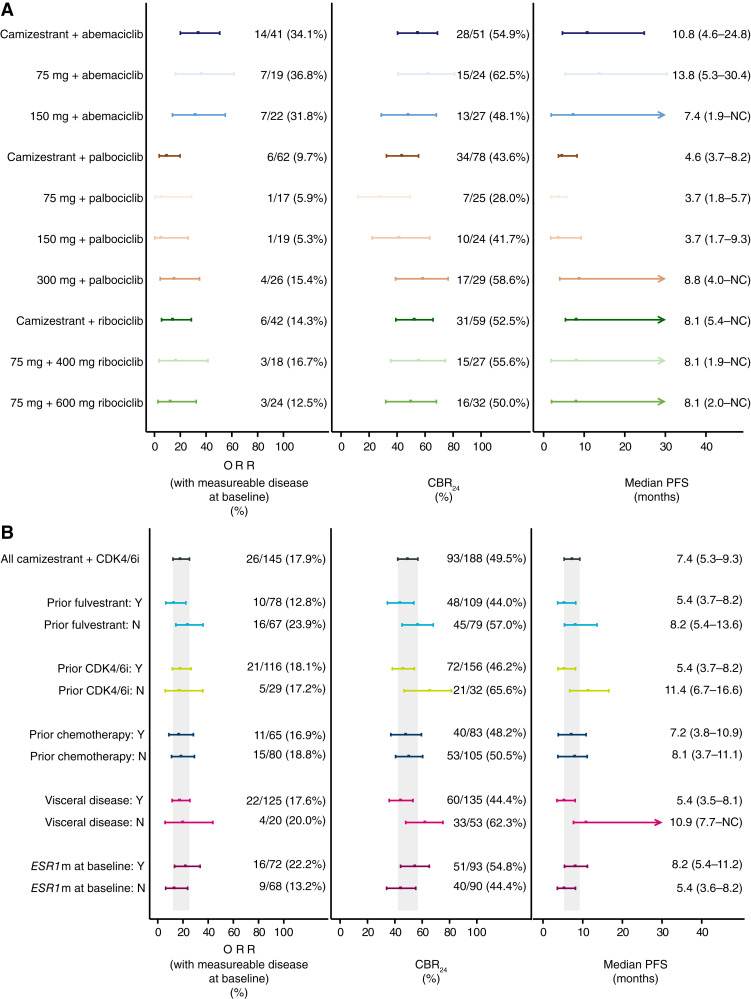
ORR, CBR_24_, and median PFS for (**A**) camizestrant in combination with abemaciclib, palbociclib, or ribociclib; (**B**) subgroup analysis pooled across all camizestrant + CDK4/6i combinations. NC is represented by an arrowed line in the plots and is not representative of the data. N, no; NC, not calculated; Y, yes. Data in % represent 95% CI.

A summary of objective response rate (ORR), clinical benefit rate at 24 weeks (CBR_24_), and median PFS for camizestrant and each CDK4/6i combination is shown in Fig. 2A. For patients who received camizestrant with abemaciclib, palbociclib, and ribociclib, the confirmed ORRs with measurable disease at baseline were 34.1% (14/41), 9.7% (6/62), and 14.3% (6/42), respectively; the CBR_24_ was 54.9% (28/51), 43.6% (34/78), and 52.5% (31/59); and the median PFS was 10.8 months [95% confidence intervals (CI): 4.6–24.8], 4.6 months (95% CI, 3.7–8.2), and 8.1 months (95% CI, 5.4–NC).

Across all camizestrant and CDK4/6i combination arms, the ORR with measurable disease at baseline, CBR_24_, and median PFS were 17.9% (26/145), 49.5% (93/188), and 7.4 months (95% CI, 5.3–9.3). Antitumor activity was also observed in patients with prior fulvestrant treatment, prior CDK4/6i treatment, with or without evidence of disease harboring *ESR1*m at baseline, and with visceral disease, including liver metastases (Fig. 2B). Similar outcomes were observed for those patients treated with camizestrant 75 mg and all CDK4/6i combinations (Supplementary Fig. S4).

### *ESR1*m ctDNA dynamics

**Figure 3.**
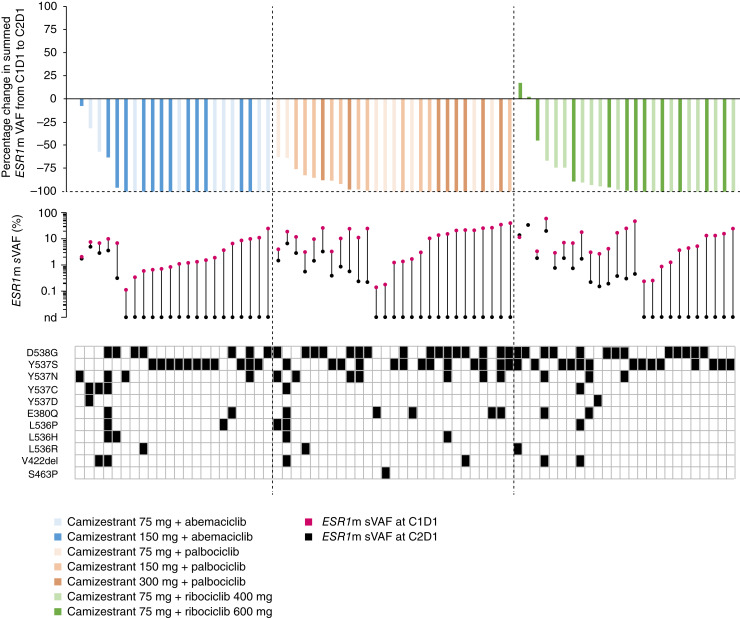
*ESR1*m ctDNA dynamics for each CDK4/6i + camizestrant combination. (Top) waterfall plot showing percentage change in *ESR1*m summed VAF at C2D1 compared with C1D1 in each evaluable patient. (Middle) vector plot showing absolute *ESR1*m ctDNA change. The dots indicate the summed VAF observed in the C1D1 sample (magenta) and the C2D1 sample (black). (Bottom) tile plot of *ESR1*m identified in each patient by ctDNA. *ESR1*m was defined as a mutation that gives rise to one of the following amino acid changes: E380Q, V422del, S463P, L536H/P/R, Y537C/D/N/S, or D538G. C1D1, cycle 1, day 1; C2D1, cycle 2, day 1; nd, not detected; sVAF, summed variant allele frequency; VAF, variant allele frequency.

The effect of camizestrant in combination with each CDK4/6i on *ESR1*m ctDNA was assessed in patients in whom *ESR1*m was detected at baseline (cycle 1, day 1), with follow-up (cycle 2, day 1) samples collected and successfully analyzed. A ≥50% reduction from baseline in summed *ESR1*m variant allele frequency was observed in 91% (20/22) of patients treated with camizestrant plus abemaciclib, 100% (27/27) of patients treated with camizestrant plus palbociclib, and 88% (22/25) of patients treated with camizestrant plus ribociclib. Furthermore, ctDNA clearance to undetectable levels at cycle 2, day 1 was observed in 77% (17/22) of patients treated with camizestrant plus abemaciclib, 59% (16/27) of patients treated with camizestrant plus palbociclib, and 44% (11/25) of patients treated with camizestrant plus ribociclib (Fig. 3). These reductions were seen across all *ESR1*m variants tested, including D538G and Y537S (Supplementary Fig. S5).

## Discussion

These results from SERENA-1 demonstrate that camizestrant has a well-tolerated safety profile with encouraging clinical activity when combined with the CDK4/6i abemaciclib, palbociclib, or ribociclib. PK data for camizestrant 75 mg (the phase III dose) in combination with abemaciclib and palbociclib were broadly consistent with camizestrant as a monotherapy. Increased exposure of camizestrant in combination with ribociclib was observed, more consistent with that of camizestrant 150 mg; however, given the safety profile observed, this was not associated with any clinically relevant effects. All CDK4/6i exposures remained consistent with published steady-state PK data, indicating no clinically meaningful drug–drug interactions (43–46).

Overall, the safety profile for each CDK4/6i administered in combination with camizestrant was consistent with their known profiles as monotherapy and in combination with other ETs. The safety profile of camizestrant when dosed in combination with each of the CDK4/6i was also consistent with its monotherapy profile. These observations, in addition to there being few instances of AEs leading to dose reduction or discontinuation of camizestrant, demonstrate that camizestrant is well tolerated, with no additive toxicity when combined with any of these three CDK4/6i.

Evidence of antitumor activity was observed for camizestrant 75 mg combined with abemaciclib, palbociclib, and ribociclib, including in patients with prior fulvestrant treatment, prior CDK4/6i treatment, with or without detectable *ESR1*m at baseline, and with visceral disease, including liver metastases.

Given that the patient population in SERENA-1 was heavily pretreated, with 83% of patients having previously received CDK4/6i and 55% having previously received palbociclib, the median PFS for camizestrant 75 mg in combination with abemaciclib [13.8 months (95% CI, 5.3, 30.4)], with ribociclib [8.1 months (5.4, NC)], or with palbociclib [3.7 months (1.8, 5.7)] was particularly notable. These data, together with the improved clinical benefit rates, support the combination of camizestrant with all three CDK4/6i.

Furthermore, a reduction of at least 50% in summed *ESR1*m variant allele frequency was observed in most patients with available data across all combinations. Clearance of *ESR1*m to undetectable levels was also observed in almost two thirds of patients, with reductions in all *ESR1*m variants tested, including D538G and Y537S, the most common mutations associated with ET resistance (47). These data demonstrate encouraging antitumor activity for all camizestrant and CDK4/6i combinations.

This study has some limitations common to phase I oncology studies. The study population was relatively small and heavily pretreated for advanced disease, which may limit the ability to draw definitive conclusions on dose–response and may confound the efficacy assessments, because of the inherent heterogeneity, affecting the overall robustness of the trial.

In terms of PK, some of the data (e.g., elimination half-life or AUC_infinity_) for camizestrant in this study are likely to be underestimated because PK was only sampled up to 24 hours after dose, making it difficult to fully characterize the terminal half-life. Indeed, data from a combined, unpublished population PK analysis of SERENA-1 data (48) and a healthy volunteer study (NCT04546347) suggest that the half-life is likely to be longer, at 20 to 23 hours.

Key strengths of this study include the dose-escalation and -expansion format, which effectively determined the maximum tolerated dose (MTD) of camizestrant when combined with each of the CDK4/6i evaluated. Other parts of SERENA-1 investigated camizestrant in combination with everolimus (49) or with capivasertib (50). The multipart design of the trial also enabled results to progressively inform dose optimization of later parts and enhanced our understanding of safety-related outcomes. Furthermore, comprehensive ECG assessments conducted across all combinations enabled characterization of HR change and onset and offset rates, providing important temporal data on key cardiac parameters.

During preparation of this article, the phase III SERENA-6 trial (NCT04964934) met its primary endpoint and data have been published (34). Switching to camizestrant with continuation of CDK4/6i guided by emergence of *ESR1*m during first-line therapy, ahead of disease progression, demonstrated a statistically significant and clinically meaningful improvement in PFS versus continuing aromatase inhibitor + CDK4/6i in patients with HR+, HER2− advanced breast cancer. PFS benefit was consistent across the CDK4/6i (abemaciclib, palbociclib or ribociclib), and the camizestrant safety profile observed was consistent with the previous SERENA trial program data, including that reported here. Camizestrant is under evaluation across several other phase III randomized clinical trials in patients with ER+, HER2− breast cancer. SERENA-4 (NCT04711252) is evaluating camizestrant and palbociclib versus anastrozole and palbociclib as upfront first-line therapy for ER+, HER2− advanced breast cancer, while in the adjuvant setting, CAMBRIA-2 (NCT05952557) compares camizestrant with/without abemaciclib with standard-of-care ET with/without abemaciclib in patients who are starting adjuvant ET and CAMBRIA-1 (NCT05774951) compares camizestrant monotherapy with standard-of-care ET in patients after at least 2 years of standard adjuvant ET.

### Conclusions

These data from the multipart SERENA-1, phase I study in patients with ER+, HER2− advanced breast cancer demonstrate that camizestrant 75 mg (phase III dose) has a tolerable safety profile when administered in combination with the CDK4/6i abemaciclib, palbociclib, and ribociclib. The patient population in this study had received extensive lines of prior therapy, including CDK4/6i, fulvestrant, and chemotherapies, and there was no selection for endocrine sensitivity. Despite this, encouraging clinical activity, including evidence of *ESR1*m ctDNA clearance, was observed for camizestrant in combination with each CDK4/6i, with no evidence of clinically relevant drug–drug interactions.

## Supplementary Material

Supplementary Figure S1 CONSORT diagrams

Supplementary Figure S2 Time course of heart rate reductions and QTcF

Supplementary Figure S3 Abemaciclib, palbociclib, and ribociclib concentrations at steady state

Supplementary Figure S4 ORR, CBR24, and median PFS for subgroup analysis across pooled camizestrant 75 mg combination arms

Supplementary Figure S5 Percentage change in ESR1m variants

Supplementary Table S1 Study representativeness

Supplementary Table S2 Safety summary of camizestrant

Supplementary Table S3 Adverse event profile for camizestrant

Supplementary Table S4 Camizestrant PK parameters

## Data Availability

Data underlying the findings described in this article may be obtained in accordance with AstraZeneca’s data sharing policy described at https://astrazenecagrouptrials.pharmacm.com/ST/Submission/Disclosure. Requests for data from studies listed on the Vivli platform can be submitted via www.vivli.org. For studies not available on the Vivli platform, data access requests can be made via: https://vivli.org/members/enquiries-about-studies-not-listed-on-the-vivli-platform/. Additional information specific to AstraZeneca can be found on their dedicated Vivli member page: https://vivli.org/ourmember/astrazeneca/.
