# Supplementary material for: Camizestrant in Combination with Three Globally Approved CDK4/6 Inhibitors in Women with ER+, HER2− Advanced Breast Cancer: Results from SERENA-1
Source: Clin Cancer Res. 2025 Aug 11;31(20):4244–54. doi: 10.1158/1078-0432.CCR-25-1198 (PMC12521909; doi:10.1158/1078-0432.CCR-25-1198)
Supplement: Supplementary Figure S1 — CONSORT diagrams [file ccr-25-1198_supplementary_figure_s1_suppfs1.docx]

**Supplementary Figure S1:** CONSORT diagram for camizestrant in combination with abemaciclib (Parts G/H), palbociclib (Parts C/D), or ribociclib (Parts K/L) parts

1. Camizestrant in combination with abemaciclib


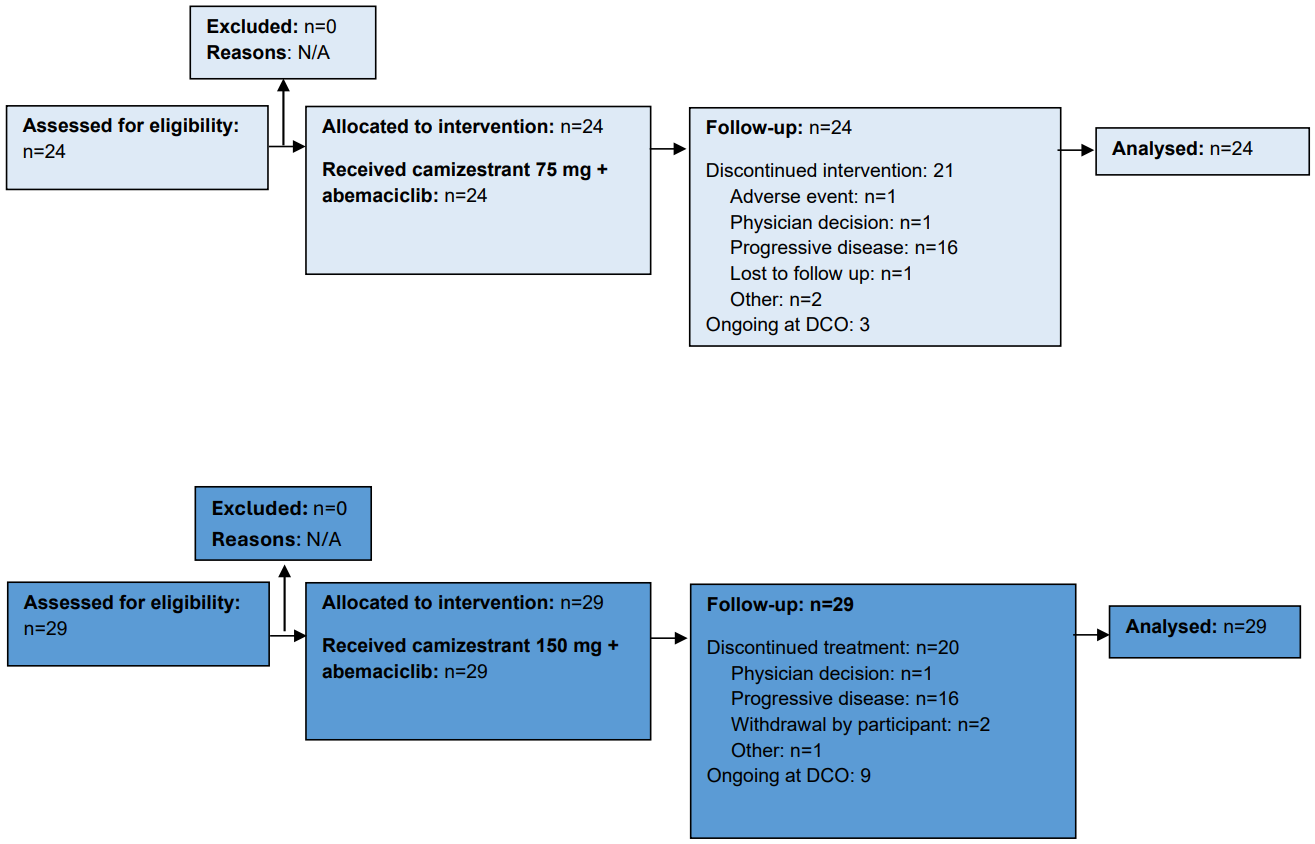


1. Camizestrant in combination with palbociclib


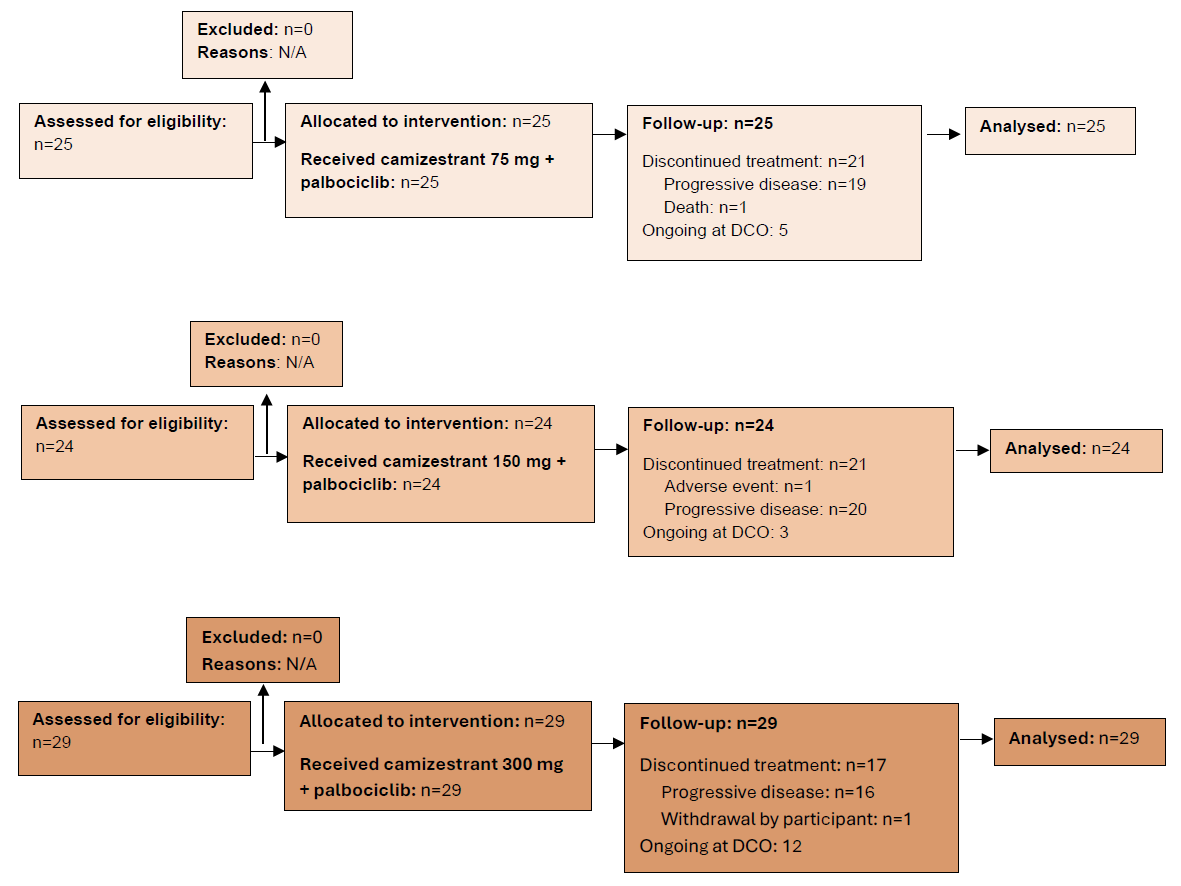


1. Camizestrant in combination with ribociclib


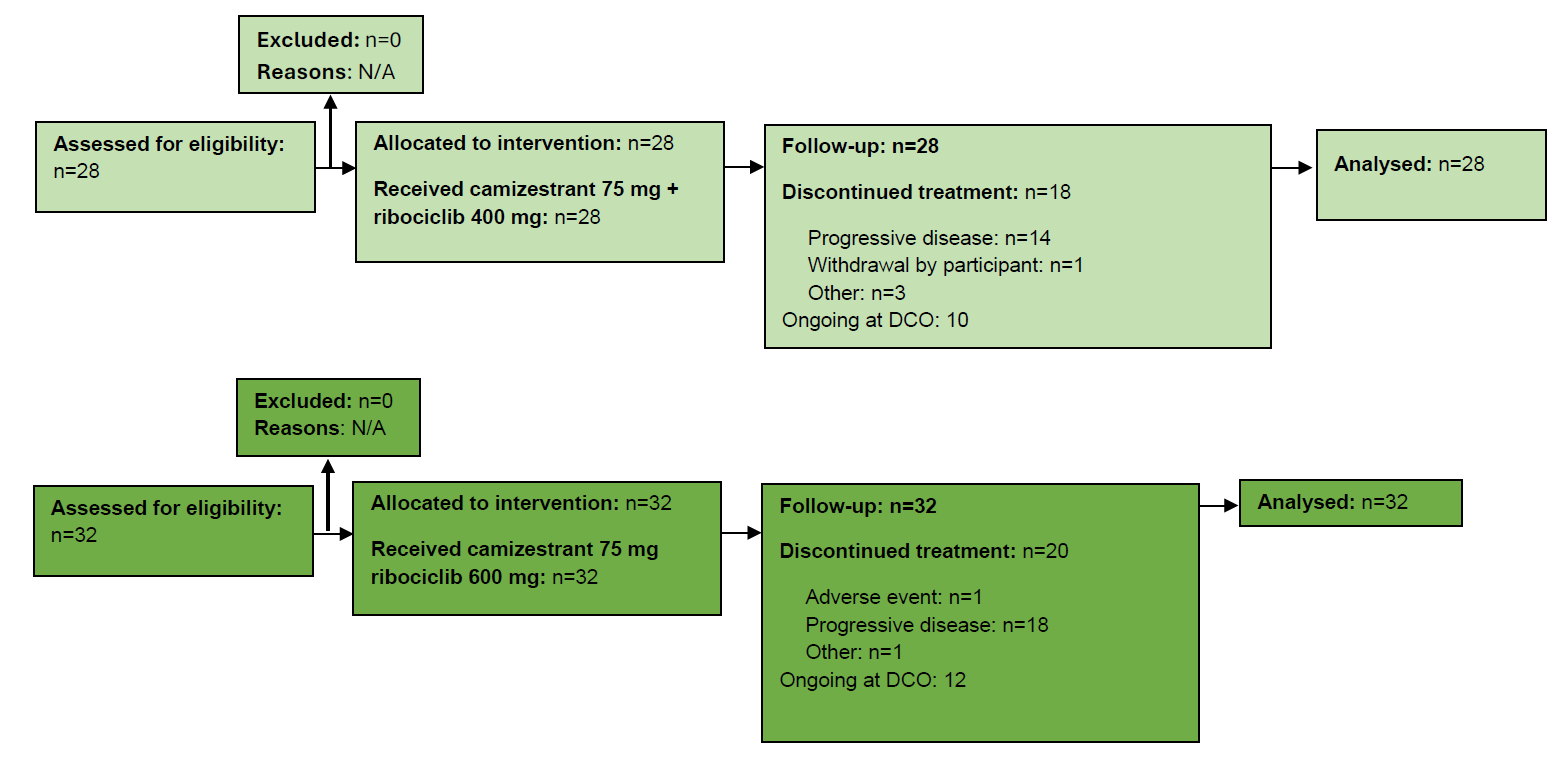


DCO, data cut off; NA, not applicable.
