# Supplementary material for: Camizestrant in Combination with Three Globally Approved CDK4/6 Inhibitors in Women with ER+, HER2− Advanced Breast Cancer: Results from SERENA-1
Source: Clin Cancer Res. 2025 Aug 11;31(20):4244–54. doi: 10.1158/1078-0432.CCR-25-1198 (PMC12521909; doi:10.1158/1078-0432.CCR-25-1198)
Supplement: Supplementary Figure S2 — Time course of heart rate reductions and QTcF [file ccr-25-1198_supplementary_figure_s2_suppfs2.docx]

**Supplementary Figure S2:** Time course of heart rate reductions and QTcF in patients treated with camizestrant 75 mg combined with either abemaciclib, palbociclib, or ribociclib

**
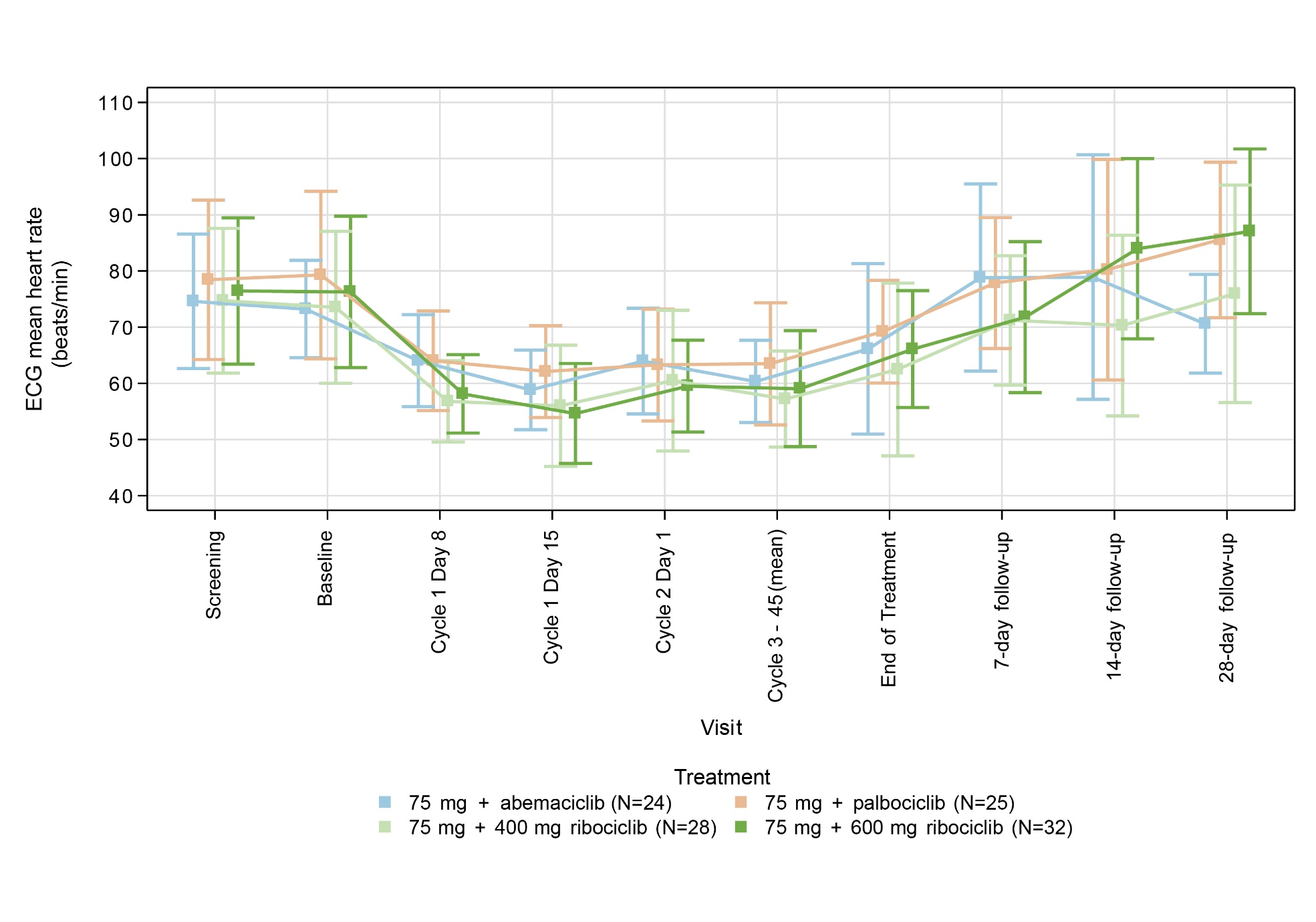
A)**

**
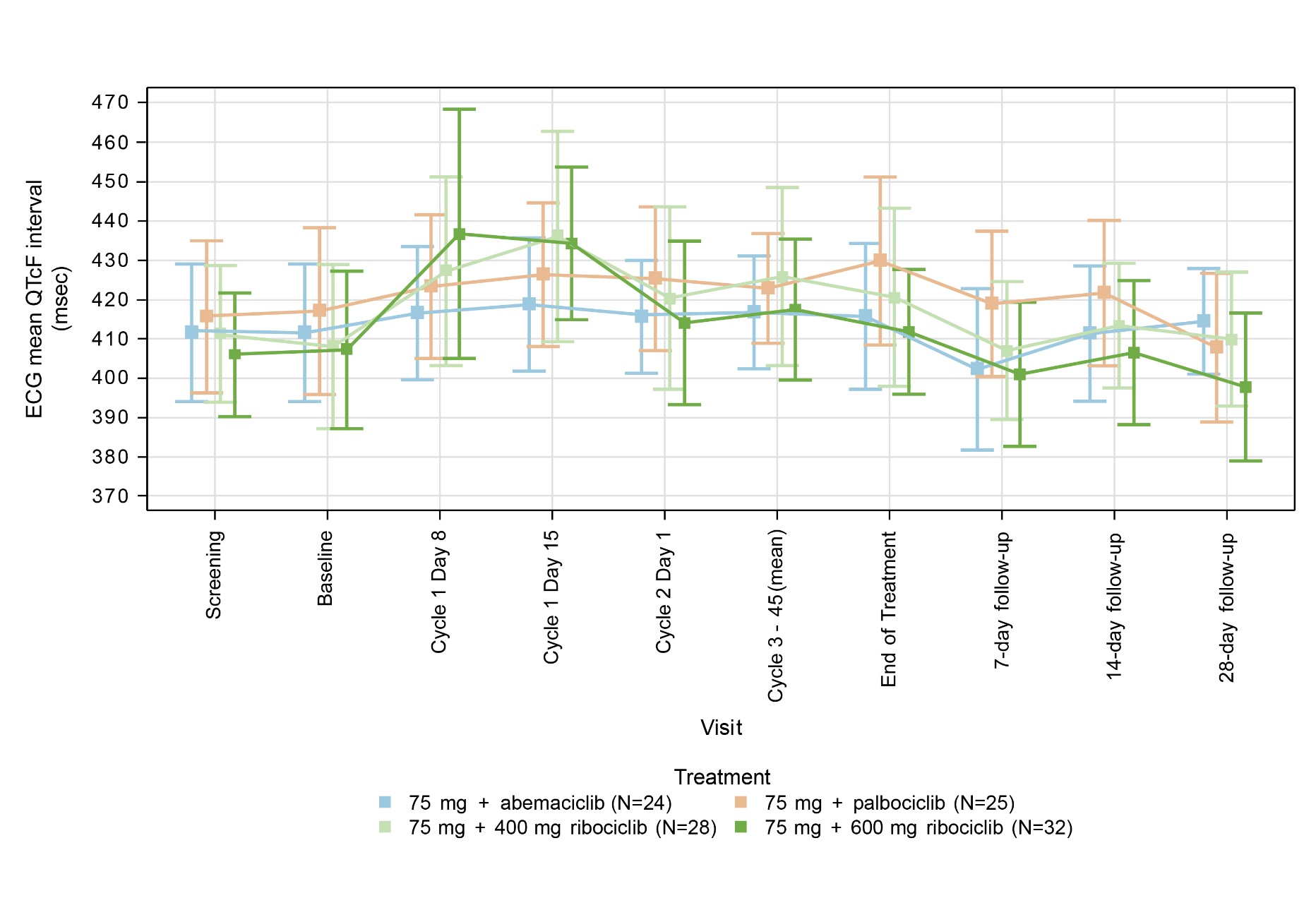
B)**

ECG, electrocardiogram; min, minute; N, number of participants; QTcF, QT interval with Fridericia’s formula correction.
