# Supplementary material for: Camizestrant in Combination with Three Globally Approved CDK4/6 Inhibitors in Women with ER+, HER2− Advanced Breast Cancer: Results from SERENA-1
Source: Clin Cancer Res. 2025 Aug 11;31(20):4244–54. doi: 10.1158/1078-0432.CCR-25-1198 (PMC12521909; doi:10.1158/1078-0432.CCR-25-1198)
Supplement: Supplementary Figure S3 — Abemaciclib, palbociclib, and ribociclib concentrations at steady state [file ccr-25-1198_supplementary_figure_s3_suppfs3.docx]

**Supplementary Figure S3:** Abemaciclib, palbociclib, and ribociclib concentrations at steady state (Cycle 1 Day 15) when in combination with camizestrant. **A)** Abemaciclib exposure (150 mg BID) exposure in combination with camizestrant 75 and 150 mg overlaid on a popPK model simulation of abemaciclib monotherapy; **B)** palbociclib (125 mg QD, 21 days on/7 days off) exposure in combination with camizestrant 75, 150, and 300 mg overlaid on a popPK model simulation of palbociclib 125 mg; **C)** ribociclib (QD 400 or 600 mg 21-days on/7-days off) exposure in combination with camizestrant 75 mg overlaid on a monotherapy popPK model simulation of ribociclib 400 and 600 mg.

**
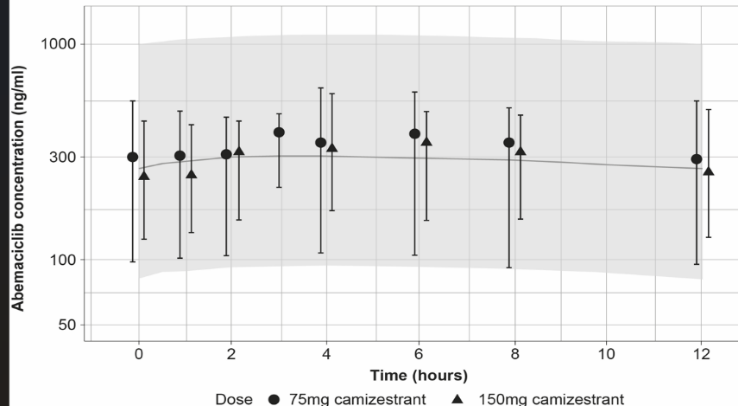
A)**

**
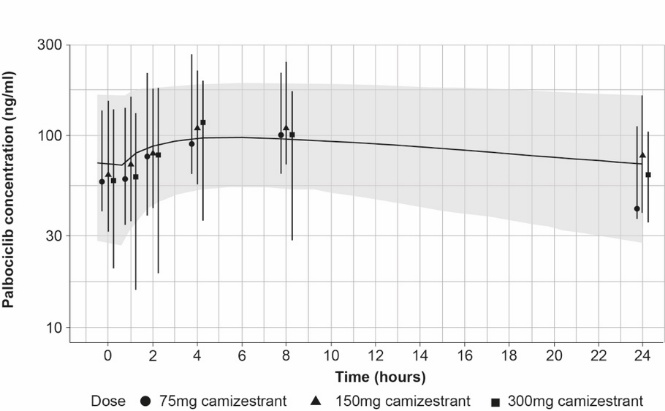
B)**

**
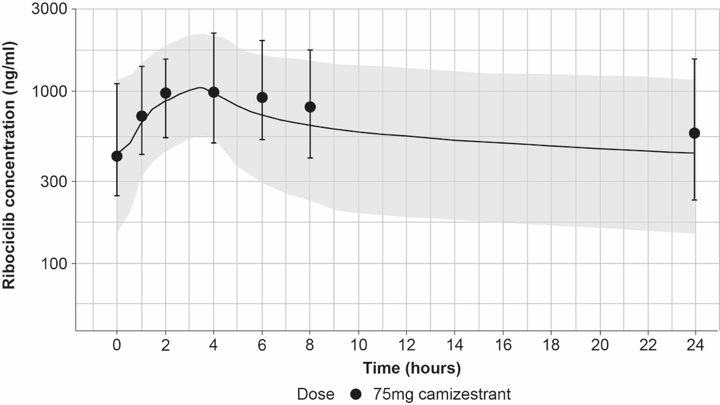

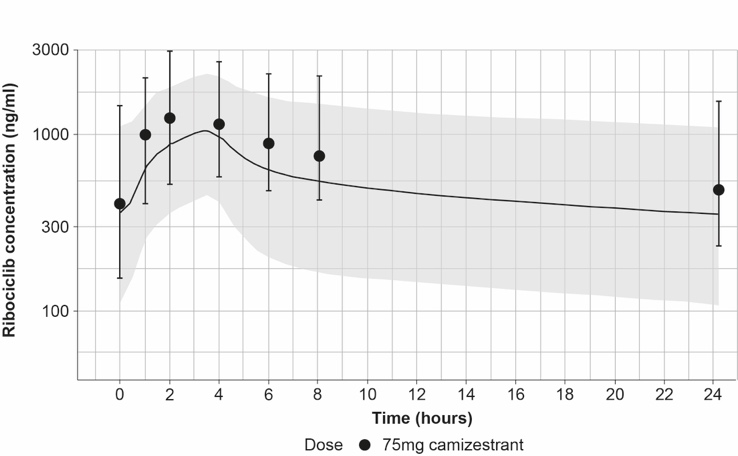
C)**

Ribociclib 400 mg

Ribociclib 600 mg

Median PK observations; error bars: 2.5th and 97.5th percentiles of the data; solid black line: median of popPK simulations (N = 1000); shadow: 95% prediction interval of popPK simulations.

BID, two times a day/bis in die; popPK, population pharmacokinetic.
