# Supplementary material for: Camizestrant in Combination with Three Globally Approved CDK4/6 Inhibitors in Women with ER+, HER2− Advanced Breast Cancer: Results from SERENA-1
Source: Clin Cancer Res. 2025 Aug 11;31(20):4244–54. doi: 10.1158/1078-0432.CCR-25-1198 (PMC12521909; doi:10.1158/1078-0432.CCR-25-1198)
Supplement: Supplementary Figure S4 — ORR, CBR24, and median PFS for subgroup analysis across pooled camizestrant 75 mg combination arms [file ccr-25-1198_supplementary_figure_s4_suppfs4.docx]

**Supplementary Figure S4:** ORR, CBR_24_, and median PFS for subgroup analysis across pooled camizestrant 75 mg combination arms


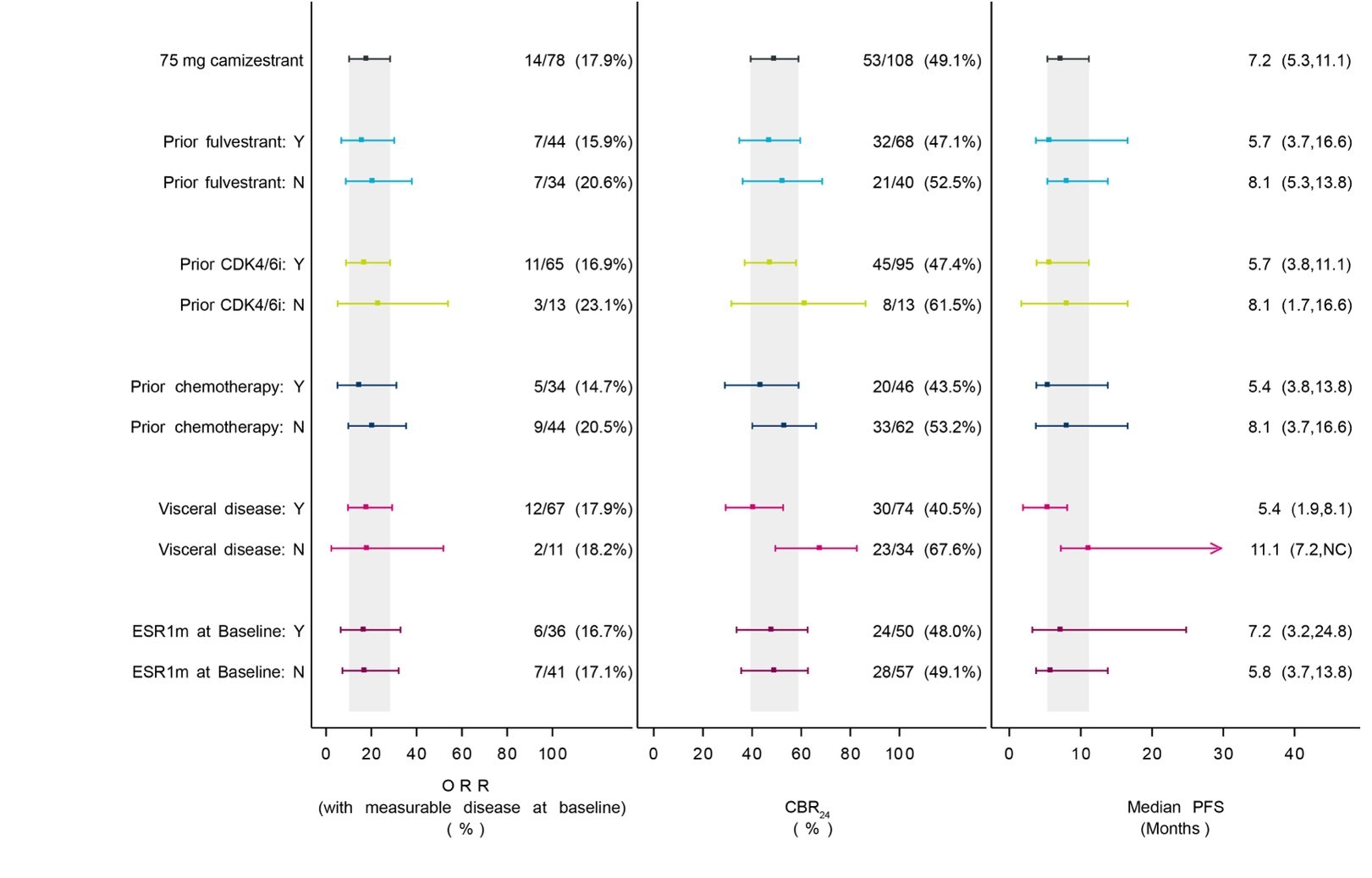


CBR_24_, clinical benefit rate at 24 weeks; CDK4/6i, cyclin-dependent kinase 4/6 inhibitor; *ESR1*, estrogen receptor 1 gene; NC: not calculated; ORR, overall response rate; PFS, progression-free survival.
