# Supplementary material for: Camizestrant in Combination with Three Globally Approved CDK4/6 Inhibitors in Women with ER+, HER2− Advanced Breast Cancer: Results from SERENA-1
Source: Clin Cancer Res. 2025 Aug 11;31(20):4244–54. doi: 10.1158/1078-0432.CCR-25-1198 (PMC12521909; doi:10.1158/1078-0432.CCR-25-1198)
Supplement: Supplementary Figure S5 — Percentage change in ESR1m variants [file ccr-25-1198_supplementary_figure_s5_suppfs5.docx]

**Supplementary Figure S5:** Percentage change in *ESR1*m variants from C1D1 to C2D1 in patients treated with for camizestrant in combination with abemaciclib, palbociclib, or ribociclib. Maximum increase limited to 100% for presentation purposes.


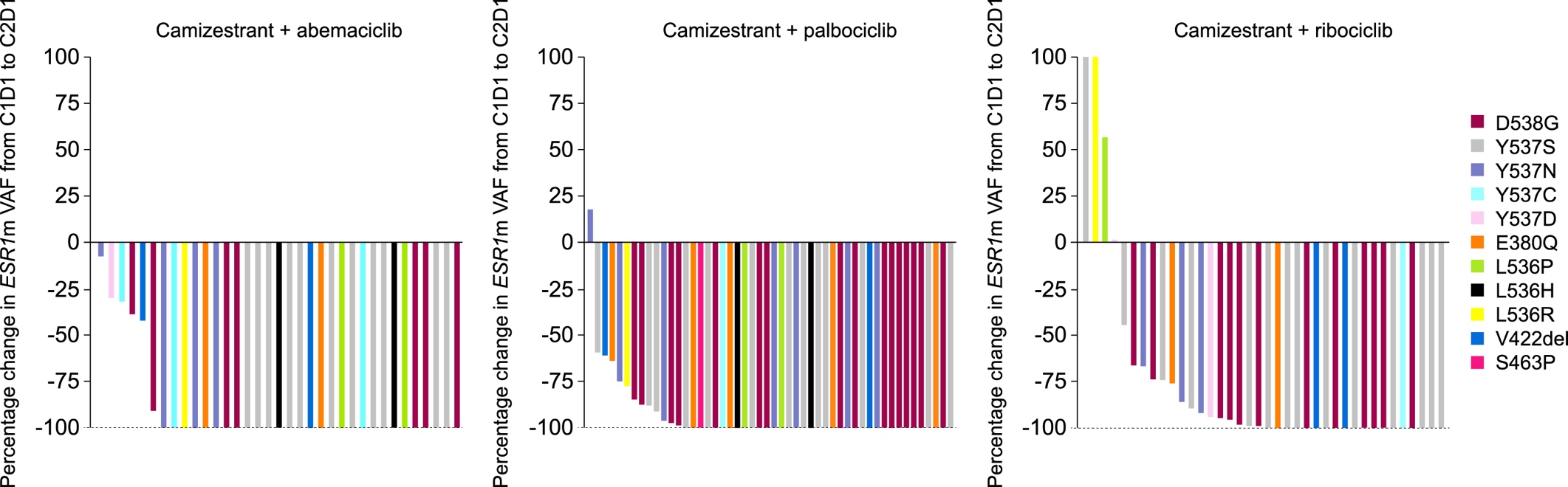


C1D1, Cycle 1 Day 1; C2D1, Cycle 2 Day 1; *ESR1*m*,* estrogen receptor-1 mutation; VAF, variant allele frequency.
