# Supplementary material for: Camizestrant in Combination with Three Globally Approved CDK4/6 Inhibitors in Women with ER+, HER2− Advanced Breast Cancer: Results from SERENA-1
Source: Clin Cancer Res. 2025 Aug 11;31(20):4244–54. doi: 10.1158/1078-0432.CCR-25-1198 (PMC12521909; doi:10.1158/1078-0432.CCR-25-1198)
Supplement: Supplementary Table S1 — Study representativeness [file ccr-25-1198_supplementary_table_s1_suppts1.docx]

**Supplementary tables**

**Supplementary Table S1.** Study representativeness

| **Population characteristic** | **Description** |
| --- | --- |
| Cancer type(s)/subtype(s)/stage(s)/condition | ER+/HER2− advanced breast cancer. |
| **Considerations related to:** | |
| Sex | Breast cancer patients are predominantly female with less than 1% of patients who are male.^2^ |
| Age | As of 2019, the median age at initial breast cancer diagnosis was 62 years, and the median age for diagnosis of advanced breast cancer was 69 years.^4,39^ |
| Race/ethnicity | Breast cancer incidence rate by ethnicity in the United States between 2010-2019 per 100,000 was:^4^  133.7 White women  127.8 Black women  111.3 American Indian/Alaska Native women, 101.3 Asian/Pacific Islander women  99.2 Hispanic women |
| Geography | Breast cancer is the second most common cancer globally, with over 2.3 million new cases reported in 2022 alone.^39^ In the USA, UK and Western Europe, breast cancer incidence was 95.9, 94.0 and 89.8 per 100,000 females in 2022; these regions are in the top four highest for breast cancer incidence worldwide.^38,40^ |
| Other considerations | Globally, the incidence of advanced/metastatic breast cancer is variable; however, it is estimated that approximately 20% of early-stage breast cancer patients will go on to develop advanced or metastatic diseases. HR+ breast cancer, including estrogen-receptor positive ER+ disease, is the most common type, accounting for approximately 68–75% of cases.^1,3,4,41^ |
| Overall representativeness of this study | Participants for SERENA-1 were recruited from the UK, Spain, and the USA. All participants in the SERENA-1 trial were female. The median age in SERENA-1 across all parts ranged from 55–61 years. Black women were underrepresented throughout the parts of the trial presented here. The population recruited to these parts of SERENA-1 where the combination of camizestrant and abemaciclib, palbociclib or ribociclib was assessed is considered broadly representative of the advanced breast cancer population where these combinations are under Phase 3 assessment (SERENA-4 [NCT04711252] and SERENA-6 [NCT04964934]). |

CDK4/6i: cyclin-dependent kinase 4/6 inhibitor; ER+: Endocrine receptor positive; ET: endocrine therapy; HER2−: human epidermal growth factor receptor 2-negative; HR+: Hormone receptor-positive.
