# Supplementary material for: Camizestrant in Combination with Three Globally Approved CDK4/6 Inhibitors in Women with ER+, HER2− Advanced Breast Cancer: Results from SERENA-1
Source: Clin Cancer Res. 2025 Aug 11;31(20):4244–54. doi: 10.1158/1078-0432.CCR-25-1198 (PMC12521909; doi:10.1158/1078-0432.CCR-25-1198)
Supplement: Supplementary Table S2 — Safety summary of camizestrant [file ccr-25-1198_supplementary_table_s2_suppts2.docx]

**Supplementary Table S2.** Safety summary of camizestrant in combination with abemaciclib, palbociclib, or ribociclib

| **N (%)** | **Camizestrant 75 mg + abemaciclib (n=24)** | **Camizestrant 150 mg + abemaciclib (n=29)** | **Camizestrant 75 mg + palbociclib (n=25)** | **Camizestrant 150 mg + palbociclib (n=24)** | **Camizestrant 300 mg + palbociclib (n=29)** | **Camizestrant 75 mg + ribociclib 400 mg (n=28)** | **Camizestrant 75 mg + ribociclib 600 mg (n=32)** |
| --- | --- | --- | --- | --- | --- | --- | --- |
| Mean treatment duration, months (SD) | 15.3 (13.3) | 8.1 (6.7) | 4.8 (3.9) | 6.5 (5.7) | 8.0 (5.7) | 6.5 (4.3) | 6.1 (4.1) |
| Any AE | 24 (100) | 29 (100) | 24 (96.0) | 24 (100) | 29 (100) | 28 (100) | 32 (100) |
| Any AE causally related to camizestrant | 21 (87.5) | 27 (93.1) | 15 (60.0) | 20 (83.3) | 28 (96.6) | 25 (89.3) | 29 (90.6) |
| Any AE causally related to CDK4/6i | 24 (100) | 28 (96.6) | 21 (84.0) | 23 (95.8) | 24 (82.8) | 25 (89.3) | 31 (96.9) |
| Any AE causally related to both camizestrant and CDK4/6i | 17 (70.8) | 18 (62.1) | 8 (32.0) | 12 (50.0) | 15 (51.7) | 18 (64.3) | 17 (53.1) |
| Any grade ≥3 AE | 20 (83.3) | 18 (62.1) | 19 (76.0) | 17 (70.8) | 18 (62.1) | 12 (42.9) | 22 (68.8) |
| Any grade ≥3 AE causally related to camizestrant | 4 (16.7) | 5 (17.2) | 0 | 1 (4.2) | 0 | 1 (3.6) | 6 (18.8) |
| Any grade ≥3 AE causally related to CDK4/6i | 16 (66.7) | 11 (37.9) | 14 (56.0) | 16 (66.7) | 17 (58.6) | 6 (21.4) | 22 (68.8) |
| Any grade ≥3 AE causally related to both camizestrant and CDK4/6i | 4 (16.7) | 3 (10.3) | 0 | 1 (4.2) | 0 | 1 (3.6) | 6 (18.8) |
| Any AE with outcome of death | 0 | 0 | 1 (4.0) | 1 (4.2) | 0 | 0 | 0 |
| Any AE with outcome of death causally related to camizestrant | 0 | 0 | 0 | 0 | 0 | 0 | 0 |
| Any AE with outcome of death causally related to CDK4/6i | 0 | 0 | 0 | 0 | 0 | 0 | 0 |
| Any AE with outcome of death causally related to both camizestrant and CDK4/6i | 0 | 0 | 0 | 0 | 0 | 0 | 0 |
| Any SAE (including AE with death) | 6 (25.0) | 9 (31.0) | 4 (16.0) | 4 (16.7) | 1 (3.4) | 4 (14.3) | 5 (15.6) |
| Any SAE (including AE with death) causally related to camizestrant | 1 (4.2) | 3 (10.3) | 0 | 0 | 0 | 0 | 1 (3.1) |
| Any SAE (including AE with death) causally related to CDK4/6i | 4 (16.7) | 5 (17.2) | 0 | 1 (4.2) | 1 (3.4) | 0 | 1 (3.1) |
| Any SAE (including AE with death) causally related to both camizestrant and CDK4/6i | 1 (4.2) | 2 (6.9) | 0 | 0 | 0 | 0 | 1 (3.1) |
| Any AE leading to discontinuation of camizestrant | 1 (4.2) | 0 | 0 | 1 (4.2) | 0 | 0 | 1 (3.1) |
| Any AE leading to dose reduction of camizestrant | 0 | 3 (10.3) | 0 | 1 (4.2) | 5 (17.2) | 0 | 0 |
| Any AE leading to dose interruption of camizestrant | 13 (54.2) | 18 (62.1) | 3 (12.0) | 8 (33.3) | 7 (24.1) | 11 (39.3) | 13 (40.6) |
| Any AE leading to discontinuation of CDK4/6i | 1 (4.2) | 1 (3.4) | 0 | 2 (8.3) | 2 (6.9) | 1 (3.6) | 1 (3.1) |
| Any AE leading to dose reduction of CDK4/6i | 15 (62.5) | 13 (44.8) | 7 (28.0) | 5 (20.8) | 10 (34.5) | 4 (14.3) | 13 (40.6) |
| Any AE leading to dose interruption of CDK4/6i | 19 (79.2) | 22 (75.9) | 13 (52.0) | 18 (75.0) | 19 (65.5) | 13 (46.4) | 25 (78.1) |

AE, adverse event; CDK4/6i, cyclin-dependent kinase 4/6 inhibitor; n, number of participants; SAE, serious adverse event; SD, standard deviation.
