# Supplementary material for: Camizestrant in Combination with Three Globally Approved CDK4/6 Inhibitors in Women with ER+, HER2− Advanced Breast Cancer: Results from SERENA-1
Source: Clin Cancer Res. 2025 Aug 11;31(20):4244–54. doi: 10.1158/1078-0432.CCR-25-1198 (PMC12521909; doi:10.1158/1078-0432.CCR-25-1198)
Supplement: Supplementary Table S3 — Adverse event profile for camizestrant [file ccr-25-1198_supplementary_table_s3_suppts3.docx]

**Supplementary Table S3:** Adverse event profile for camizestrant in combination with abemaciclib, palbociclib, or ribociclib (irrespective of causality); adverse events reported in ≥20% of patients by grade*

| **N (%)** | **C75 mg + A (*n*=24)** | | **C 150 mg + A (*n*=29)** | | **C 75 mg + P (*n*=25)** | | **C 150 mg + P (*n*=24)** | | **C 300 mg + P (*n*=29)** | | **C 75 mg + R 400 mg (*n*=28)** | | **C 75 mg + R 600 mg (*n*=32)** | |
| --- | --- | --- | --- | --- | --- | --- | --- | --- | --- | --- | --- | --- | --- | --- |
|  | **Any grade** | **Grade ≥3** | **Any grade** | **Grade ≥3** | **Any grade** | **Grade ≥3** | **Any grade** | **Grade ≥3** | **Any grade** | **Grade ≥3** | **Any grade** | **Grade ≥3** | **Any grade** | **Grade ≥3** |
| Abdominal Pain | 5 (20.8) [3,1,1,0,0] | 1 (4.2) | 5 (17.2) [3,2,0,0,0] | 0 | 2 (8.0) [1,0,1,0,0] | 1 (4.0) | 5 (20.8) [2,3,0,0,0] | 0 | 2 (6.9) [1,1,0,0,0] | 0 | 5 (17.9) [4,0,1,0,0] | 1 (3.6) | 1 (3.1) [1,0,0,0,0] | 0 |
| Alanine Aminotransferase Increased | 5 (20.8) [2,0,3,0,0] | 3 (12.5) | 4 (13.8) [1,0,3,0,0] | 3 (10.3) | 0 | 0 | 1 (4.2) [1,0,0,0,0] | 0 | 2 (6.9) [2,0,0,0,0] | 0 | 2 (7.1) [2,0,0,0,0] | 0 | 5 (15.6) [3,1,1,0,0] | 1 (3.1) |
| Anaemia | 4 (16.7) [1,3,0,0,0] | 0 | 8 (27.6) [3,3,2,0,0] | 2 (6.9) | 5 (20.0) [2,2,1,0,0] | 1 (4.0) | 8 (33.3) [0,7,1,0,0] | 1 (4.2) | 9 (31.0) [5,3,1,0,0] | 1 (3.4) | 5 (17.9) [2,3,0,0,0] | 0 | 4 (12.5) [2,2,0,0,0] | 0 |
| Arthralgia | 6 (25.0) [3,3,0,0,0] | 0 | 1 (3.4) [1,0,0,0,0] | 0 | 3 (12.0) [3,0,0,0,0] | 0 | 2 (8.3) [1,1,0,0,0] | 0 | 6 (20.7) [5,1,0,0,0] | 0 | 7 (25.0) [4,3,0,0,0] | 0 | 4 (12.5) [1,2,1,0,0] | 1 (3.1) |
| Aspartate Aminotransferase Increased | 6 (25.0) [5,0,1,0,0] | 1 (4.2) | 6 (20.7) [3,0,3,0,0] | 3 (10.3) | 0 | 0 | 4 (16.7) [4,0,0,0,0] | 0 | 1 (3.4) [0,1,0,0,0] | 0 | 3 (10.7) [3,0,0,0,0] | 0 | 6 (18.8) [4,1,1,0,0] | 1 (3.1) |
| Asthenia | 6 (25.0) [2,3,1,0,0] | 1 (4.2) | 9 (31.0) [8,1,0,0,0] | 0 | 3 (12.0) [3,0,0,0,0] | 0 | 8 (33.3) [5,3,0,0,0] | 0 | 10 (34.5) [5,5,0,0,0] | 0 | 8 (28.6) [7,1,0,0,0] | 0 | 6 (18.8) [4,2,0,0,0] | 0 |
| Back Pain | 5 (20.8) [1,4,0,0,0] | 0 | 6 (20.7) [3,2,1,0,0] | 1 (3.4) | 1 (4.0) [0,0,1,0,0] | 1 (4.0) | 2 (8.3) [2,0,0,0,0] | 0 | 4 (13.8) [4,0,0,0,0] | 0 | 2 (7.1) [1,1,0,0,0] | 0 | 5 (15.6) [3,1,1,0,0] | 1 (3.1) |
| Blood Creatinine Increased | 6 (25.0) [4,2,0,0,0] | 0 | 9 (31.0) [6,2,1,0,0] | 1 (3.4) | 2 (8.0) [2,0,0,0,0] | 0 | 1 (4.2) [1,0,0,0,0] | 0 | 2 (6.9) [2,0,0,0,0] | 0 | 4 (14.3) [3,1,0,0,0] | 0 | 4 (12.5) [4,0,0,0,0] | 0 |
| Bradycardia | 2 (8.3) [2,0,0,0,0] | 0 | 5 (17.2) [5,0,0,0,0] | 0 | 0 | 0 | 4 (16.7) [4,0,0,0,0] | 0 | 7 (24.1) [6,1,0,0,0] | 0 | 1 (3.6) [1,0,0,0,0] | 0 | 2 (6.3) [2,0,0,0,0] | 0 |
| Constipation | 2 (8.3) [2,0,0,0,0] | 0 | 7 (24.1) [7,0,0,0,0] | 0 | 3 (12.0) [2,1,0,0,0] | 0 | 5 (20.8) [3,2,0,0,0] | 0 | 4 (13.8) [4,0,0,0,0] | 0 | 1 (3.6) [1,0,0,0,0] | 0 | 2 (6.3) [1,1,0,0,0] | 0 |
| Cough | 9 (37.5) [9,0,0,0,0] | 0 | 4 (13.8) [4,0,0,0,0] | 0 | 0 | 0 | 4 (16.7) [3,1,0,0,0] | 0 | 2 (6.9) [2,0,0,0,0] | 0 | 7 (25.0) [7,0,0,0,0] | 0 | 6 (18.8) [5,1,0,0,0] | 0 |
| Covid-19 | 6 (25.0) [6,0,0,0,0] | 0 | 2 (6.9) [1,1,0,0,0] | 0 | 0 | 0 | 1 (4.2) [0,0,0,0,1] | 1 (4.2) | 2 (6.9) [1,1,0,0,0] | 0 | 2 (7.1) [1,1,0,0,0] | 0 | 1 (3.1) [0,1,0,0,0] | 0 |
| Decreased Appetite | 9 (37.5) [3,6,0,0,0] | 0 | 7 (24.1) [4,2,1,0,0] | 1 (3.4) | 3 (12.0) [2,1,0,0,0] | 0 | 4 (16.7) [2,1,1,0,0] | 1 (4.2) | 1 (3.4) [1,0,0,0,0] | 0 | 2 (7.1) [1,1,0,0,0] | 0 | 1 (3.1) [1,0,0,0,0] | 0 |
| Diarrhoea | 21 (87.5) [12,7,2,0,0] | 2 (8.3) | 24 (82.8) [16,6,2,0,0] | 2 (6.9) | 3 (12.0) [3,0,0,0,0] | 0 | 1 (4.2) [1,0,0,0,0] | 0 | 1 (3.4) [1,0,0,0,0] | 0 | 8 (28.6) [6,0,2,0,0] | 2 (7.1) | 8 (25.0) [6,2,0,0,0] | 0 |
| Dizziness | 6 (25.0) [6,0,0,0,0] | 0 | 3 (10.3) [1,0,2,0,0] | 2 (6.9) | 2 (8.0) [2,0,0,0,0] | 0 | 4 (16.7) [4,0,0,0,0] | 0 | 2 (6.9) [2,0,0,0,0] | 0 | 2 (7.1) [2,0,0,0,0] | 0 | 5 (15.6) [4,1,0,0,0] | 0 |
| Dry Eye | 5 (20.8) [3,2,0,0,0] | 0 | 3 (10.3) [3,0,0,0,0] | 0 | 1 (4.0) [1,0,0,0,0] | 0 | 3 (12.5) [3,0,0,0,0] | 0 | 6 (20.7) [6,0,0,0,0] | 0 | 1 (3.6) [0,1,0,0,0] | 0 | 2 (6.3) [2,0,0,0,0] | 0 |
| Dysgeusia | 5 (20.8) [4,0,1,0,0] | 1 (4.2) | 5 (17.2) [5,0,0,0,0] | 0 | 2 (8.0) [1,1,0,0,0] | 0 | 1 (4.2) [0,1,0,0,0] | 0 | 1 (3.4) [1,0,0,0,0] | 0 | 0 | 0 | 0 | 0 |
| Dyspnoea | 2 (8.3) [1,1,0,0,0] | 0 | 5(17.2) [1,2,2,0,0] | 2 (6.9) | 2 (8.0) [2,0,0,0,0] | 0 | 3 (12.5) [1,1,0,1,0] | 1 (4.2) | 1 (3.4) [0,1,0,0,0] | 0 | 7 (25.0) [5,1,1,0,0] | 1 (3.6) | 5 (15.6) [2,2,0,1,0] | 1 (3.1) |
| Electrocardiogram Qt Prolonged | 0 | 0 | 3 (10.3) [0,1,2,0,0] | 2 (6.9) | 1 (4.0) [1,0,0,0,0] | 0 | 1 (4.2) [0,1,0,0,0] | 0 | 2 (6.9) [1,1,0,0,0] | 0 | 6 (21.4) [3,2,1,0,0] | 1 (3.6) | 5 (15.6) [2,1,1,1,0] | 2 (6.3) |
| Fatigue | 11 (45.8) [3,6,2,0,0] | 2 (8.3) | 8 (27.6) [3,5,0,0,0] | 0 | 5 (20.0) [4,1,0,0,0] | 0 | 6 (25.0) [3,2,1,0,0] | 1 (4.2) | 5 (17.2) [3,2,0,0,0] | 0 | 5 (17.9) [2,3,0,0,0] | 0 | 7 (21.9) [4,3,0,0,0] | 0 |
| Haemoglobin Decreased | 2 (8.3) [0,2,0,0,0] | 0 | 6 (20.7) [5,1,0,0,0] | 0 | 0 | 0 | 0 | 0 | 0 | 0 | 3 (10.7) [1,2,0,0,0] | 0 | 5 (15.6) [2,3,0,0,0] | 0 |
| Headache | 7 (29.2) [5,2,0,0,0] | 0 | 6 (20.7) [6,0,0,0,0] | 0 | 2 (8.0) [2,0,0,0,0] | 0 | 1 (4.2) [0,1,0,0,0] | 0 | 2 (6.9) [2,0,0,0,0] | 0 | 3 (10.7) [2,1,0,0,0] | 0 | 0 [0,0,0,0,0] | 0 |
| Hypertension | 5 (20.8) [1,1,3,0,0] | 3 (12.5) | 3 (10.3) [1,2,0,0,0] | 0 | 2 (8.0) [0,0,2,0,0] | 2 (8.0) | 0 | 0 | 1 (3.4) [0,1,0,0,0] | 0 | 5 (17.9) [1,3,1,0,0] | 1 (3.6) | 5 (15.6) [3,2,0,0,0] | 0 |
| Lacrimation Increased | 5 (20.8) [3,2,0,0,0] | 0 | 0 [0,0,0,0,0] | 0 | 0 [0,0,0,0,0] | 0 | 1 (4.2) [1,0,0,0,0] | 0 | 1 (3.4) [1,0,0,0,0] | 0 | 0 [0,0,0,0,0] | 0 | 0 | 0 |
| NT-proBNP Increased | 2 (8.3) [2,0,0,0,0] | 0 | 5 (17.2) [5,0,0,0,0] | 0 | 0 | 0 | 0 [0,0,0,0,0] | 0 | 1 (3.4) [1,0,0,0,0] | 0 | 2 (7.1) [1,1,0,0,0] | 0 | 7 (21.9) [7,0,0,0,0] | 0 |
| Nausea | 11 (45.8) [5,6,0,0,0] | 0 | 13 (44.8) [8,4,1,0,0] | 1 (3.4) | 4 (16.0) [3,1,0,0,0] | 0 | 7 (29.2) [5,1,1,0,0] | 1 (4.2) | 7 (24.1) [7,0,0,0,0] | 0 | 10 (35.7) [9,0,1,0,0] | 1 (3.6) | 14 (43.8) [12,2,0,0,0] | 0 |
| Neutropenia | 7 (29.2) [0,2,5,0,0] | 5 (20.8) | 6 (20.7) [0,5,1,0,0] | 1 (3.4) | 20 (80.0) [0,6,11,3,0] | 14 (56.0) | 20 (83.3) [4,0,15,1,0] | 16 (66.7) | 22 (75.9) [0,4,18,0,0] | 18 (62.1) | 9 (32.1) [3,3,3,0,0] | 3 (10.7) | 17 (53.1) [2,1,13,1,0] | 14 (43.8) |
| Neutrophil Count Decreased | 6 (25.0) [2,0,4,0,0] | 4 (16.7) | 5 (17.2) [1,0,4,0,0] | 4 (13.8) | 0 [0,0,0,0,0] | 0 | 0 [0,0,0,0,0] | 0 | 0 | 0 | 4 (14.3) [2,0,2,0,0] | 2 (7.1) | 5 (15.6) [0,0,5,0,0] | 5 (15.6) |
| Photopsia | 4 (16.7) [4,0,0,0,0] | 0 | 6 (20.7) [5,1,0,0,0] | 0 | 4 (16.0) [4,0,0,0,0] | 0 | 6 (25.0) [5,1,0,0,0] | 0 | 6 (20.7) [6,0,0,0,0] | 0 | 11 (39.3) [11,0,0,0,0] | 0 | 11 (34.4) [11,0,0,0,0] | 0 |
| Platelet Count Decreased | 6 (25.0) [6,0,0,0,0] | 0 | 2 (6.9) [1,0,1,0,0] | 1 (3.4) | 4 (16.0) [4,0,0,0,0] | 0 | 5 (20.8) [3,0,2,0,0] | 2 (8.3) | 8 (27.6) [3,1,3,1,0] | 4 (13.8) | 0 | 0 | 0 | 0 |
| Pruritus | 5 (20.8) [3,2,0,0,0] | 0 | 0 | 0 | 1 (4.0) [1,0,0,0,0] | 0 | 1 (4.2) [1,0,0,0,0] | 0 | 1 (3.4) [1,0,0,0,0] | 0 | 4 (14.3) [3,1,0,0,0] | 0 | 2 (6.3) [1,1,0,0,0] | 0 |
| Sinus Bradycardia | 5 (20.8) [5,0,0,0,0] | 0 | 12 (41.4) [11,1,0,0,0] | 0 | 4 (16.0) [4,0,0,0,0] | 0 | 5 (20.8) [5,0,0,0,0] | 0 | 12 (41.4) [11,1,0,0,0] | 0 | 11 (39.3) [10,1,0,0,0] | 0 | 10 (31.3) [10,0,0,0,0] | 0 |
| Urinary Tract Infection | 8 (33.3) [1,7,0,0,0] | 0 | 6 (20.7) [1,5,0,0,0] | 0 | 2 (8.0) [0,2,0,0,0] | 0 | 3 (12.5) [0,3,0,0,0] | 0 | 2 (6.9) [1,1,0,0,0] | 0 | 3 (10.7) [1,2,0,0,0] | 0 | 2 (6.3) [2,0,0,0,0] | 0 |
| Visual Impairment | 7 (29.2) [6,1,0,0,0] | 0 | 9 (31.0) [8,1,0,0,0] | 0 | 5 (20.0) [5,0,0,0,0] | 0 | 8 (33.3) [8,0,0,0,0] | 0 | 5 (17.2) [4,1,0,0,0] | 0 | 5 (17.9) [5,0,0,0,0] | 0 | 4 (12.5) [4,0,0,0,0] | 0 |
| Vitreous Floaters | 1 (4.2) [1,0,0,0,0] | 0 | 3 (10.3) [3,0,0,0,0] | 0 | 0 | 0 | 2 (8.3) [2,0,0,0,0] | 0 | 0 | 0 | 2 (7.1) [2,0,0,0,0] | 0 | 7 (21.9) [7,0,0,0,0] | 0 |
| Vomiting | 8 (33.3) [6,2,0,0,0] | 0 | 11 (37.9) [8,2,1,0,0] | 1 (3.4) | 3 (12.0) [1,2,0,0,0] | 0 | 6 (25.0) [6,0,0,0,0] | 0 | 3 (10.3) [3,0,0,0,0] | 0 | 6 (21.4) [4,1,1,0,0] | 1 (3.6) | 7 (21.9) [5,2,0,0,0] | 0 |
| Weight Decreased | 5 (20.8) [4,1,0,0,0] | 0 | 3 (10.3) [1,1,1,0,0] | 1 (3.4) | 0 | 0 | 1 (4.2) [0,1,0,0,0] | 0 | 0 | 0 | 0 | 0 | 0 | 0 |

*Reported in ≥20% of patients in any cohort at any Grade.
A, abemaciclib; C, camizestrant; NT-proBNP, N-Terminal Prohormone Brain Natriuretic Peptide Increased; P, palbociclib; R, ribociclib.
CTCAE grades are reported as the number of patients with Grade 1, Grade 2, Grade 3, Grade 4, Grade 5. N (%); G1, G2, G3, G4, G5.
