# Supplementary material for: Camizestrant in Combination with Three Globally Approved CDK4/6 Inhibitors in Women with ER+, HER2− Advanced Breast Cancer: Results from SERENA-1
Source: Clin Cancer Res. 2025 Aug 11;31(20):4244–54. doi: 10.1158/1078-0432.CCR-25-1198 (PMC12521909; doi:10.1158/1078-0432.CCR-25-1198)
Supplement: Supplementary Table S4 — Camizestrant PK parameters [file ccr-25-1198_supplementary_table_s4_suppts4.docx]

**Supplementary Table S4:** Camizestrant PK parameters at Cycle 1 Day 15 in combination with abemaciclib, palbociclib, or ribociclib

| **Camizestrant**  **(mg)** | **Camizestrant monotherapy** | | | **Abemaciclib** | | | **Palbociclib** | | | **Ribociclib (400 mg)** | | | | **Ribociclib (600 mg)** | | |
| --- | --- | --- | --- | --- | --- | --- | --- | --- | --- | --- | --- | --- | --- | --- | --- | --- |
|  | **C_max_**  **(ng/mL)** | **t_max_**  **(h)** | **AUC_tau_**  **(h.ng/mL)** | **C_max_**  **(ng/mL)** | **t_max_**  **(h)** | **AUC_tau_**  **(h.ng/mL)** | **C_max_**  **(ng/mL)** | **t_max_**  **(h)** | **AUC_tau_**  **(h.ng/mL)** | **C_max_**  **(ng/mL)** | **t_max_**  **(h)** | **AUC_tau_**  **(h.ng/mL)** | **C_max_**  **(ng/mL)** | | **t_max_**  **(h)** | **AUC_tau_**  **(h.ng/mL)** |
| **75** | 64.7 | 3.72 | 862.3 | 85.4 | 4 | 1124 | 93.9 | 4 | 1197 | 153.6 | 3.6 | 2277 | 179.2 | | 4 | 2749 |
|  | (49.2) | (2–8) | (46.5) | (41.2) | (1–6) | (41.2) | (55.7) | (1–4) | (48.7) | (38.3) | (1–6) | (44) | (50.8) | | (1–8) | (54.9) |
|  | [22] | [22] | [22] | [16] | [16] | [15] | [24] | [24] | [24] | [23] | [23] | [22] | [22] | | [22] | [22] |
| **150** | 165.6 | 2.17 | 2162 | 158.8 | 4 | 2050 | 189.0 | 3.92 | 2419 | – | – | – | – | | – | – |
|  | (36.1) | (1–24) | (38.9) | (33.3) | (1–24) | (24.9) | (39.5) | (1–8) | (45.4) |  |  |  |  | |  |  |
|  | [23] | [23] | [23] | [18] | [18] | [18] | [21] | [21] | [21] |  |  |  |  | |  |  |
| **300** | 375.8 | 3 | 4784 | – | – | – | 385.5 | 4 | 4936 | – | – | – | – | | – | – |
|  | (40.6) | (2–7) | (37.7) |  |  |  | (63.4) | (1–8) | (64.6) |  |  |  |  | |  |  |
|  | [28] | [28] | [26] |  |  |  | [25] | [25] | [25] |  |  |  |  | |  |  |

Data are presented with the exception of Geomean (% geometric CV) [n] presented with the exception of t_max_ where median (min–max) [n].
